# Genetic Adaptation Associated with Genome-Doubling in Autotetraploid *Arabidopsis arenosa*

**DOI:** 10.1371/journal.pgen.1003093

**Published:** 2012-12-20

**Authors:** Jesse D. Hollister, Brian J. Arnold, Elisabeth Svedin, Katherine S. Xue, Brian P. Dilkes, Kirsten Bomblies

**Affiliations:** 1 Department of Organismic and Evolutionary Biology, Harvard University, Cambridge, Massachusetts, United States of America; 2 Department of Horticulture and Landscape Architecture, Purdue University, West Lafayette, Indiana, United States of America; 3 Molecular Evolutionary Genetics, Interdisciplinary Life Science Program, Purdue University, West Lafayette, Indiana, United States of America; University of Georgia, United States of America

## Abstract

Genome duplication, which results in polyploidy, is disruptive to fundamental biological processes. Genome duplications occur spontaneously in a range of taxa and problems such as sterility, aneuploidy, and gene expression aberrations are common in newly formed polyploids. In mammals, genome duplication is associated with cancer and spontaneous abortion of embryos. Nevertheless, stable polyploid species occur in both plants and animals. Understanding how natural selection enabled these species to overcome early challenges can provide important insights into the mechanisms by which core cellular functions can adapt to perturbations of the genomic environment. *Arabidopsis arenosa* includes stable tetraploid populations and is related to well-characterized diploids *A. lyrata* and *A. thaliana.* It thus provides a rare opportunity to leverage genomic tools to investigate the genetic basis of polyploid stabilization. We sequenced the genomes of twelve *A. arenosa* individuals and found signatures suggestive of recent and ongoing selective sweeps throughout the genome. Many of these are at genes implicated in genome maintenance functions, including chromosome cohesion and segregation, DNA repair, homologous recombination, transcriptional regulation, and chromatin structure. Numerous encoded proteins are predicted to interact with one another. For a critical meiosis gene, *ASYNAPSIS1*, we identified a non-synonymous mutation that is highly differentiated by cytotype, but present as a rare variant in diploid *A. arenosa*, indicating selection may have acted on standing variation already present in the diploid. Several genes we identified that are implicated in sister chromatid cohesion and segregation are homologous to genes identified in a yeast mutant screen as necessary for survival of polyploid cells, and also implicated in genome instability in human diseases including cancer. This points to commonalities across kingdoms and supports the hypothesis that selection has acted on genes controlling genome integrity in *A. arenosa* as an adaptive response to genome doubling.

## Author Summary

Duplication of an entire set of chromosomes is a dramatic mutation disruptive to core cellular functions. Genome duplication and the genomic instability that generally follows can cause problems with fertility and viability, and in mammals is associated with cancer and spontaneous abortion. Yet, established polyploids occur naturally in both plants and animals. How do these organisms overcome these early problems and ultimately stabilize? The genetic basis of the adaptive response to polyploidy has remained almost completely unknown. We took advantage of modern genomic approaches to gain insight into this using a stable polyploid plant, *Arabidopsis arenosa*. We found evidence of selection in genes that control core genome maintenance processes. These overlap with genes or functions shown in yeast to be necessary for survival of polyploid cells and in humans implicated in cancer. Our results identify genes controlling core genome maintenance functions that may have undergone compensatory adaptation after genome doubling.

## Introduction

The duplication of an entire set of chromosomes is a game-changing mutation. Whole-genome duplication (WGD) may create challenges for basic biological functions. For example, the regulation of gene expression, chromosome segregation, chromatin structure, and the maintenance of cellular homeostasis with altered cell size may be perturbed by duplicating an entire set of chromosomes [1]–[8]. That WGD can be challenging to organisms across kingdoms is evidenced by observations of dysfunction in very different contexts, such as reduced fertility observed in many newly formed plant autopolyploids, and mitotic instability in polyploid cancer cells [1], [5], [9]. Despite potential roadblocks, polyploid species are abundant in nature and genome doubling has been implicated in speciation and adaptive radiations [10]. Polyploids are especially well known among plants, but also occur in a diverse array of animals, including vertebrates [11].

The short-term consequences of WGD have been extensively studied in both natural and synthetic polyploids, especially in plants. These studies indicate that chromosome structural changes and rearrangements are common following WGD, as are abnormalities in mitosis and meiosis; in some cases changes in gene expression have also been observed (e.g. see [1]–[8]). These observations support the idea that polyploidy can pose challenges to aspects of gene regulation, chromosome organization and chromosome segregation. A yeast mutant screen indicates that some of these challenges are common across kingdoms. Genes encoding proteins implicated in the maintenance of genome integrity, including homologous recombination, DNA repair, sister chromatid cohesion and mitotic spindle function were identified as essential genes specifically in tetraploids [12].

The existence of stable, fertile polyploid species in different kingdoms demonstrates that the challenges that genome-doubled organisms may face at their inception are not insurmountable, and suggests that genome-doubled lineages should experience a period of compensatory genetic adaptation to their genome-doubled state. In sharp contrast to our understanding of the early transcriptional or genomic responses of organisms to WGD [1]–[8], very little is known about what molecular mechanisms might contribute to longer-term stabilization of polyploids or adaptation to a genome-doubled state. In plants, a single gene important for polyploid stabilization has been molecularly characterized: the homologous pairing suppressor (*Ph1*) from allohexaploid wheat. Allopolyploids like wheat have hybrid origins and carry already somewhat divergent sets of chromosomes. *Ph1* enhances meiotic pairing preferences of chromosomes for more similar (homologous) chromosomes over less similar (homeologous) ones, resulting in bivalent pairing and stable meiosis [13]. This work provides an important molecular insight into the process of meiotic stabilization in allopolyploids.

However, not all polyploids stabilize meiosis by developing pairing preferences. Autopolyploids arise from within-species genome duplications and thus carry four homologs of each chromosome [1], [3]–[5], [14]. Established autopolyploids often have cytologically diploidized meiosis (forming primarily bivalent associations), but show polysomic inheritance at genetic markers, which is possible if the chromosomes lack pairing preferences and partner randomly at meiosis [4], [5], [14]. Thus there must be at least two mechanisms by which polyploids can stabilize meiosis, one that involves enhancing pairing preferences (as is common in allopolyploids like wheat) and one that ensures bivalent formation without affecting pairing preference.

The molecular mechanisms that underlie long-term polyploid stabilization and evolution remain largely mysterious. To help fill this gap, we undertook a population genomic analysis of an established autotetraploid plant, *Arabidopsis arenosa.* This species is closely related to two sequenced *Arabidopsis* diploids: its sister taxa *A. lyrata* and the model system *A. thaliana* [15]–[17]. Like *A. lyrata*, *A. arenosa* is obligately outcrossing, and abundant throughout Europe [16], [17]. Tetraploid *A. arenosa* is cytologically diploidized, with primarily bivalent chromosome associations at meiosis [18]. We sequenced the genomes of twelve tetraploid *A. arenosa* individuals from four populations in Germany and Austria and tested for allele frequency patterns suggestive of selective sweeps. We identified 192 genes in the *A. arenosa* genome with patterns of polymorphism indicative of recent or ongoing selective sweeps. Several functional classes represented among these genes are consistent with adaptation to WGD. We provide candidate genes that will help boost our mechanistic understanding of these processes, while also suggesting new hypotheses. Similarities of the functional classes we identified with those identified in a yeast mutant study [12] indicate that at least some challenges are shared across kingdoms, and suggests that the functions targeted by selection in *A. arenosa* are especially critical in tetraploids.

## Results

### Genome analysis of *A. arenosa*

**Figure 1:**
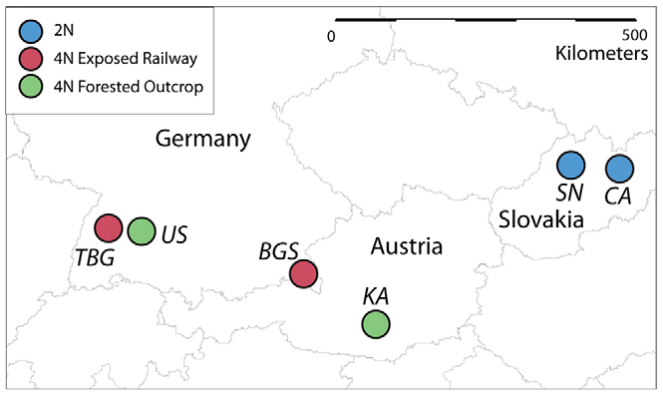
Geographic locations of *A. arenosa* populations sampled in this study. Geographic locations of sampled tetraploid populations from railways (red) and forested rock outcrops (green), and two diploid populations (blue). TBG = Triberg railway station, Germany; US = Upfinger Steige, Bad Urach, Germany; BGS = Berchtesgaden railway station, Germany; KA = Kasparstein castle, Austria; SN = Streçno castle, Slovakia; CA = Carpathian Mountains, Southern Tatras range, Slovakia. For genome sequencing, we sampled three plants each from TBG, US, BGS and KA.

We selected 12 *A. arenosa* individuals grown from seeds collected at four sites in Austria and Germany (Figure 1) for genome sequencing. Cytological and flow cytometric analyses demonstrated that *A. arenosa* populations throughout these regions are tetraploid [19], [20]. We confirmed ploidy for at least one individual from each population by flow cytometric analysis of nuclear DNA content (Figure S1), and performed testcrosses for the remainder. We aligned DNA sequence data to the publicly available reference genome of *A. lyrata* [15]. After filtering for sequence and mapping quality, overall genome coverage per sequenced individual averaged 25× across the eight *A. lyrata* chromosome scaffolds (Figure S2). We focused subsequent analyses on coding regions. We used a maximum likelihood method to infer tetraploid genotypes for each single nucleotide polymorphism (SNP) in each individual.

We generated three-species alignments with consensus sequences from all sites in the *A. arenosa* sample that had at least 4× coverage per individual, with homologs from both *A. thaliana* and *A. lyrata*. In total 26,655,179 bp were aligned, representing 20,889 homologous genes. The final dataset contains 3,148,695 segregating sites (Table 1). The average divergence of *A. arenosa* from *A. lyrata* per site was 8.7×10^−4^, 2.7×10^−4^, and 9.6×10^−4^ for synonymous, non-synonymous, and intronic positions, respectively. In addition, there were 13,634 fixed differences in *A. arenosa* consensus sequences relative to both *A. thaliana* and *A. lyrata*, distributed among 5,855 protein-coding genes, 2,147 of which contained at least one non-synonymous fixed difference relative to the *A. lyrata* reference.

**Table 1:** Polymorphism in *A. arenosa* genome data.

SNP Class	S	Watterson's θ	π
Synonymous	1,084,188	0.043 (0.042)	0.043 (0.042)
Replacement	1,061,510	0.017 (0.015)	0.013 (0.010)
Intron	1,005,295	0.043 (0.042)	0.044 (0.045)

Table 1 notes: SNP = single nucleotide polymorphism within coding regions; S = number of segregating sites (S); For Watterson's θ and for pairwise diversity (π), we report mean values with median values in parentheses.

**Figure 2:**
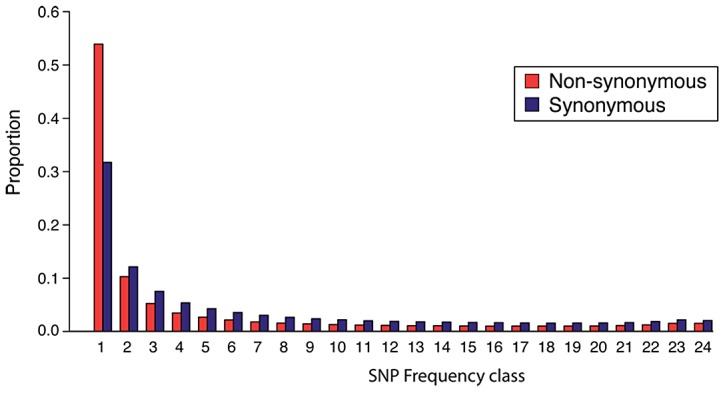
The site frequency spectrum of *A. arenosa*. Folded site frequency spectrum (SFS) of single nucleotide polymorphisms (SNPs) in *A. arenosa* protein-coding sequences. Each column indicates the abundance of SNPs that fall into a particular frequency class and columns are color-coded to indicate non-synonymous sites (red) and synonymous sites (blue). There is a significant skew toward low-frequency mutations at non-synonymous sites (red) compared to synonymous sites (dark blue) (Mann-Whitney U Test *p*<7×10^−8^).

Other studies have previously found that polymorphism in *A. arenosa* is higher than in *A. lyrata* [21], [22]. Consistent with this, we found high levels of segregating variation genome-wide in *A. arenosa* (Table 1), and synonymous site diversity approximately double that estimated for diploid *A. lyrata* [21]. This is consistent with the prediction that equilibrium genetic variation in an outcrossing autotetraploid population with tetrasomic inheritance should be approximately double that of a diploid population of similar size [23]. The site frequency spectrum (SFS) of non-synonymous SNPs showed a significant skew toward low-frequency mutations compared to the synonymous SFS (Mann-Whitney U Test *p*<7×10^−8^), consistent with widespread purifying selection (Figure 2). Importantly, the sequencing error rate we estimated from the data (0.1–0.2%; see Methods) was an order of magnitude below our estimates of Theta for all classes of sites, and the likelihood function in our genotyping algorithm explicitly accounted for errors. Thus, sequencing errors were unlikely to have contributed significantly to our estimates of diversity.

### Estimation of mode of inheritance

Inheritance can vary in tetraploids from disomic to tetrasomic. Disomic inheritance results when chromosomes have pairing partner preferences (genes thus behave as duplicates segregating two alleles each). Tetrasomic inheritance occurs in species that lack pairing preferences among the four homologous copies of each chromosome, in which case each locus segregates four alleles. Whether populations have tetrasomic or disomic inheritance has significant implications for population genetic analyses of tetraploids [3]. Therefore we investigated historic and ongoing modes of inheritance in *A. arenosa* by comparing our sequence data to simulated datasets.

We used coalescent simulations to generate expected neutral SFS and genotype frequencies under different historical scenarios and inheritance models. Our observed data did not differ significantly from simulated SFS for the tetrasomic model, but did differ from both disomic models (*p*<0.01 Mann-Whitney U test; Table S1). Similar results were obtained for inferred genotypic classes (Figure S3; Table S1). Importantly, we do not observe an excess of duplex (AAaa) genotypes, or a high number of SNPs with frequency ∼50% in the data, both of which are expected if the *A. arenosa* sample had been evolving under disomic inheritance for a significant amount of time (Figure S3). These results strongly support the hypothesis that *A. arenosa* has tetrasomic inheritance. Together with prior findings that this species has bivalent chromosome associations at meiosis [18], this places *A. arenosa* on a growing list of established tetraploids with cytologically, but not genetically diploidized meiosis [14]. Importantly, tetraploid *A. arenosa* will display patterns of polymorphism typical of a population of diploids with twice the effective size [23], [24]. Therefore, signatures of adaptive evolution are detectable using methods developed for diploids.

### Signatures of selection in *A. arenosa*

We used diploid *A. lyrata* and *A. thaliana* reference genomes [15], [25] to identify 20,265 genes that had >80% sequence identity among all three species. These genes comprise the dataset used in all analyses described below. The sampled individuals originate from four populations with distinct habitats (Figure 1). We tested for population structure or habitat-associated differentiation by pairwise *F_ST_* comparisons across the genome [26]. Overall there was low differentiation among populations. Genome wide pairwise *F_ST_* at synonymous sites ranged from 0.047 to 0.063 (Table S2), which is an order of magnitude lower than average pairwise *F_ST_* measured between populations of *A. lyrata* [22]. This suggests that *A. arenosa* lacks strong local population differentiation in this geographic region.

During the formation and early establishment of an autotetraploid, alleles that contribute to tetraploid formation or are important for the success of the tetraploid lineage should experience strong selection. To perform genome-wide tests for selection in tetraploid *A. arenosa* we identifed genes for which SFS were skewed toward high frequency derived haplotypes [27] and genes in which polymorphism was low. The two measures were uncorrelated genome-wide (R^2^ = 0.014) and together provide evidence of past selective sweeps. There were 192 genes that were both within the 5% most skewed SFS and the 5% lowest polymorphism (Table S3).

In most cases, candidate selected genes were unlinked. There were only eight instances where genes separated by less than 10 kb both showed signatures of selection. As a result, almost all potential selective sweep signatures in *A. arenosa* are sufficiently narrow to identify single candidate genes based on homology to *A. thaliana* (www.arabidopsis.org). Several gene ontology categories are over-represented among these genes (Fisher's Exact Test *p*<0.005 for each category) compared to their representation within the entire genome. These include functions related to the regulation of basal transcription, epigenetic regulation, sister chromatid cohesion, homologous recombination, DNA repair, cell cycle, cell morphogenesis and cell growth. The genes representing the most enriched categories are summarized in Table S4. We focus below on two general categories in more detail: transcriptional regulation and meiosis.

### Regulation of transcription

**Figure 3:**
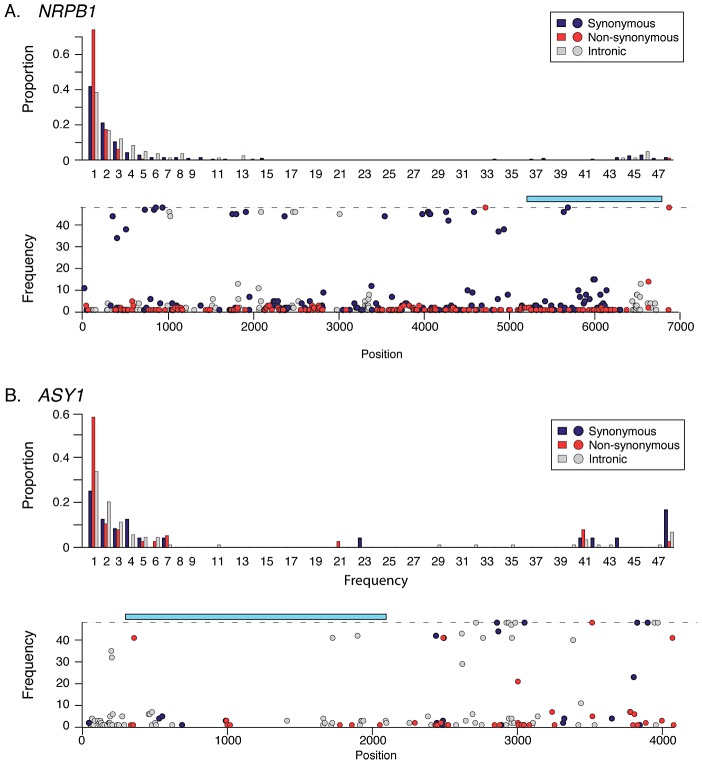
Site frequency spectra and SNP frequency for *NRPB1* and *ASY1*. (A) Polymorphism in *NRPB1*. Top graph shows unfolded SFS (top graph) relative to *A. lyrata*. Lower graph shows SNP frequencies along the gene's length relative to *A. lyrata* and *A. thaliana*. Light blue rectangle indicates region coding for C-terminal heptad repeat tail. (B) Polymorphism in *ASY1*. Top graph shows unfolded SFS (top graph) relative to *A. lyrata*. Lower graph shows SNP frequencies along the gene's length relative to *A. lyrata* and *A. thaliana*. Light blue rectangle indicates region encoding conserved HORMA domain. Non-synonymous sites are shown in red, synonymous in dark blue, and intronic sites in grey.

A “retuning” of basal transcription in response to increased cell size may be important in polyploids for maintaining a balance between expression from additional chromosome copies and altered cell size and/or nuclear membrane surface to volume ratio [1], [3]. In this light, it is intriguing that numerous genes showing indications of selection in *A. arenosa* encode proteins implicated in basal transcription, including the large subunits of two of the core DNA-dependent RNA Polymerases (Pol) II and III (Tables S3, S4). The gene encoding the large subunit of Pol II (*NRPB1*) has numerous high frequency SNP differences in *A. arenosa* relative to *A. lyrata* and *A. thaliana*. These include two fixed amino acid differences flanking either side of the highly conserved long C-terminal tail (CTD; Figure 3A). The CTD consists of a series of heptad repeats whose phosphorylation state regulates the activity of the Pol II complex [28]. In yeast, phosphorylation of the CTD is orchestrated by three cyclin dependent kinases, CDK 7, 8 and 9 [29]. A homolog of *CDK8*, *HUA ENHANCER 3* (*HEN3*) [29], also shows evidence of having undergone a selective sweep in *A. arenosa*. Two other CTD-interactors, *PRE-MRNA PROCESSING PROTEIN 40A* (*PRP40A*) and *GENERAL TRANSCRIPTION FACTOR B1* (*GTB1*) also show evidence of selective sweeps (Table S4).

In addition to the CTD-interactors, other genes encoding regulators of Pol II activity or recruitment also show signatures of selection in *A. arenosa* (Table S4). These include genes encoding core transciption factors such as two *TRANSCRIPTION FACTOR IIS* (*TFIIS*) family genes and *TBP-ASSOCIATED FACTOR 5* (*TAF5*), which encodes a subunit of TFIID. TFIID and TFIIS are general transcription factors that associate with Pol II and promote its movement during transcription [28]. We also find evidence of selection on *STRUWWELPETER* and *CENTER CITY*, which encode subunits of RNA Pol II-recruiting mediator complexes [30], [31]. Together, the signatures in these genes, as well as epigenetic regulators including genes implicated in RNA-mediated silencing, histone modification and chromatin remodeling (Table S4), suggest that a global re-tuning of transcription may have been very important in the history of *A. arenosa*.

### Meiosis

Autopolyploids also face an important handicap in meiosis: They are equipped with meiotic machinery inherited from diploid ancestors optimized over evolutionary time to segregate pairs of homologous chromosomes. That an increase to four homologs presents an obstacle is evident in newly formed tetraploids, which often show high rates of sterility due to failures of chromosome segregation in meiosis [1]–[5]. In *A. arenosa*, eight loci homologous to genes essential for meiosis fit our selective sweep criteria. These have predicted roles in chromosome synapsis, cohesion and homologous recombination (Tables S3, S4). These genes include *SISTER CHROMATID COHESION2* (*SCC2*), which encodes an adherin that loads cohesins during meiosis [32], and one of its substrates, the cohesin subunit *STRUCTURAL MAINTENANCE OF CHROMOSOMES 3* (*SMC3*) [33], [34]. *SMC5* and *SMC6a* are also among the eight meiosis-related genes that show signatures of selective sweeps. These encode proteins that function together in sister chromatid alignment, cohesion, DNA repair and homologous recombination during mitosis [35]. Recently the SMC5/6 complex was also shown to play an essential role in meiosis [36]. While sister chromatid cohesion has not previously been specifically discussed as a possible challenge for tetraploid plants, genes involved in sister chromatid cohesion were also shown to be crucial for survival of tetraploid yeast [12].

We compiled a list of 59 genes annotated in TAIR10 (www.arabidopsis.org) as playing a role in meiosis that also had clear homologs in *A. lyrata* as well as in our *A. arenosa* sample (Table S5). This set of genes showed enrichment for the signatures of positive selection. Among the 59 genes, 17 (29%) showed a significantly skewed SFS and nine showed low polymorphism in *A. arenosa* (Table S5). Eight of these genes (13.5%) were among the 192 that were in both the upper 5% tail of the CLR distribution as well as the lower 5% tail of the π/site distribution (Table S3), which is a 10-fold enrichment (Fisher's exact test *p*≪0.001). Six meiosis-related genes with skewed SFS in *A. arenosa* (top 5% genome-wide) are homologous to genes that were also identified as critical for survival in tetraploid yeast [12]. These are *RAD54, MEIOTIC RECOMBINATION 11 (MRE11), RECQ4A, TOPOISOMERASE3 (TOP3), SMC1* and *SEPARASE (ESP)* (Fisher's Exact Test p<0.001). This indicates again that fundamental aspects of chromosome biology present challenges upon genome doubling in very different species and that sister chromatid cohesion, homologous recombination and DNA repair are key shared processes.

**Figure 4:**
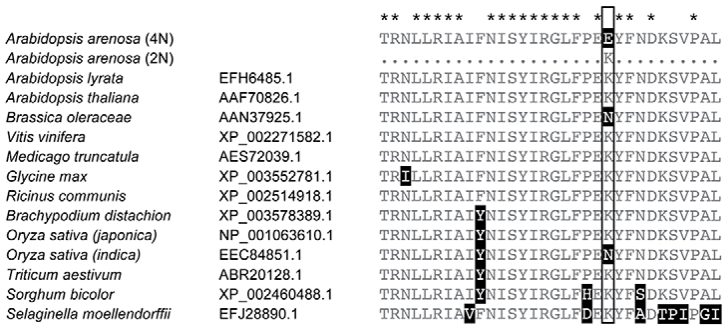
Conservation within the HORMA domain of ASY1. Alignment of a portion of the conserved HORMA domain of ASY1 with related sequences obtained from GenBank (Species names and GenBank numbers are given). Stars above the alignment indicate amino acids perfectly conserved among these sequences. The boxed amino acid position indicates one in which a derived allele (K>E) predominates in tetraploid *A. arenosa* that is rare in diploid *A. arenosa* and not found in other species reported in Genbank.

In *A. arenosa*, the chromosome synapsis gene *ASYNAPSIS1* (*ASY1*) [37] has a strongly skewed SFS, low polymorphism and an abundance of high frequency derived SNPs relative to *A. lyrata* and *A. thaliana* (Figure 3B). A high-frequency derived SNP in the tetraploid *A. arenosa* population sample of *ASY1* causes an amino acid change in the conserved HORMA domain. This alters an ancestral positively charged lysine (K) to a negatively charged glutamic acid (E) in the derived allele. We examined other *ASY1* sequences reported to date in Genbank and found that this amino acid position is conserved in a wide range of vascular plants (Figure 4). Only two other plant species have amino acid changes at this residue. Both replaced the lysine with a polar uncharged asparagine (N).

We tested whether this polymorphism is differentiated between diploid and tetraploid cytotypes within *A. arenosa* using a PCR marker. We genotyped 38 plants from two diploid populations collected from the Carpathian Mountains in Slovakia (SN and CA in Figure 1B). We found that the derived allele is present, but rare in the diploids (at a frequency of ∼4%). In sharp contrast, in tetraploid *A. arenosa*, the derived allele represents 41 of the 48 assayed sequences in our genome resequencing data (85%) and in a wider sample of 75 tetraploids from five additional populations, the derived allele has a frequency ∼90%.

### Gene interactions

We next asked whether any of the selected genes in *A. arenosa* are predicted to interact using the AtPIN database [38]. Forty-six (∼24%) of the 192 candidate selected proteins are known or predicted to interact with at least one other on the list (). Twelve genes encode products indicated in pairwise interactions. A set of four forms a small network associated with *TARGET OF RAPAMYCIN* (*TOR*) and *RAPTOR*, which regulate a variety of processes associated with cell proliferation [39]–[41]. A set of three is associated with a ubiquitin protein ligase, *UPL4* [42] (Table S6).

**Figure 5:**
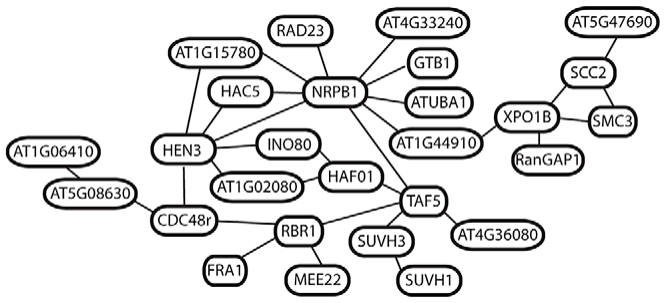
Predicted interactions among 27 putatively selected genes in *A. arenosa*. Network shows connections predicted by the AtPIN database (see methods) among selected genes in *A. arenosa*.

All of the remaining 27 genes are linked in a single network of predicted interactions, many with multiple connections per node (Figure 5). The two most connected are *NRPB1* (9 connections) and *HEN3* (6 connections). Many of the additional genes linked to these encode regulators of basal transcription, chromatin structure and cell cycle. This includes several additional interactors of the CTD tail of NRPB1, core transcription factor components such as TAF5 [28], [43], and histone modifiers implicated in the regulation of transcription, including *HISTONE ACETYLTRANSFERASE 5* and *TAF1* [28], [43], [44] (Figure 5). Shared links through nuclear-cytoplasmic trafficking via *EXPORTIN1B* connect the network surrounding *NRPB1* and *HEN3* to a small group of genes involved in regulation of chromatin structure and cohesion in meiosis, including *SMC3* and *SCC2*. None of these 27 genes are closely linked in the genome, suggesting that multiple components of this interaction network have been under selection.

## Discussion

Here we report results from a population genomic analysis in autotetraploid *A. arenosa.* We show that *A. arenosa* has high genetic diversity, little population structure, and allele and genotype frequencies consistent with a history of tetrasomic inheritance, in which four alleles segregate at each genomic locus. We identified 192 genes that exhibit two signatures of selective sweeps: reduced diversity and a SFS skewed toward high frequency derived alleles. It is important to note that our analysis could not identify loci contributing to polyploid stabilization strictly via adaptive changes in gene expression pattern, unless accompanied by a signature of selection that extended into coding regions. Identification of such loci would require comparative analysis of gene expression patterns among diploids and tetraploids, and/or analysis of sequence evolution in intergenic regions. Nevertheless, our focus on adaptive evolution within protein-coding regions allowed identification of putatively selected genes that have clear orthologs in *A. thaliana*, and for which functional information is therefore available.

This work suggests candidate genes and processes that may have been important for compensatory adaptation of *A. arenosa* to its genome-doubled state. The functional annotations of the *A. thaliana* homologs of these genes point to the modulation of fundamental biological processes, including the regulation of core transcription, epigenetic regulation, DNA repair, cell division and morphogenesis, chromosome synapsis and cohesion, homologous recombination, and chromosome segregation. Several of these categories represent functions that have been previously demonstrated or hypothesized to be problematic for neo-polyploids, but for which the mechanisms of longer-term stabilization have not been studied [1]–[5].

Several functional classes represented among candidate selected genes in *A. arenosa*, particularly chromosome cohesion, segregation and repair, show considerable overlap with genes necessary for survival specifically in polyploid yeast [12]. Moreover, six genes with SFS indicative of selection are the closest (or only) *Arabidopsis* homologs of the genes identified in the yeast screen. These are *RAD54, MRE11, SMC1, TOP3, RECQ4A,* and *ESP*. That these genes are truly fundamental in genome maintenance is also underlined by the fact that all of them have been implicated in numerous human diseases associated with genome instability, including cancer, Ataxia-Telangiectasia-like disorders, Bloom Syndrome and others, e.g. [45]–[50]. This indicates that at least some of the fundamental challenges to the maintenance of genome integrity that organisms face after genome perturbations, including whole genome duplication, are broadly shared across kingdoms. It also provides corroborative evidence that at least some of the signatures of selection in *A. arenosa* are indeed attributable to adaptation to a doubled genome.

There have been numerous studies of gene expression in response to whole genome duplication (see e.g. [2], [6]–[8]). Though most have focused on allopolyploids, several have directly compared gene expression in diploids and their autotetraploid derivates (e.g. [51]–[57]). In most cases, there is little or no overlap with the functional classes or specific genes identified in expression studies and those we identified in our study. This suggests that the genes and functional classes involved in short-term responses to genome duplication are largely distinct from those that may be under selection during longer-term polyploid evolution. There are some exceptions: In *Paspalum notatum*, gene expression changes in new polyploids occur in some of the same gene classes as those we identified here, including transcription, DNA repair and chromatin structure regulation [51]. Thus in some cases early gene expression responses do occur in genes or functional classes that may be under selection in longer-term polyploid evolution, suggesting that some of the selection acting on polyploid genomes may be a compensatory response to early shifts in gene expression. One of the genes we identified as putatively under selection in *A. arenosa*, *RAD54*, which is involved in DNA repair as well as homologous recombination [58], [59], has also been reported to be upregulated in response to genome duplication in autotetraploid *A. thaliana* [39] (though see [54]).

Another feature of the putatively selected genes in *A. arenosa* is that many are known or predicted to interact. This is especially true of genes implicated in the regulation of basal transcription. That multiple functionally connected, but unlinked genes may have experienced selective sweeps suggests that these loci either contribute incrementally to fitness through modifications of a common process or have been selected together as a functional module. Entire networks can experience selection effectively as units if epistatic interactions are synergistic and alter the selective environment for mutations at functionally related loci, allowing a larger coordinated response to selection [60], [61]. Indeed, findings in other species support the idea that genetic modules encoding networks of interacting proteins can in some circumstances respond to selection as units [60]–[65]. Whether interaction effects have driven selection on a functional module surrounding basal transcription in *A. arenosa*, or whether the polymorphisms contribute additively to a selected phenotype merits further exploration. Interestingly, in yeast it has also been noted that genes important in tetraploid survival are predicted to interact extensively [12], suggesting that this, too, may be a shared feature of polyploids across kingdoms.

Processes such as core transcription are interlinked with other cellular functions. For some genes we have identified it will be possible to clearly hypothesize what the selected function is. However, for other genes, it is less clear what function selection has acted to modulate, or if there are pleiotropic effects. For example, *GTB1*, which shows evidence of selection in *A. arenosa*, binds the C-terminal extension of Pol II and participates in regulation of Pol II processivity [28]. Thus it is reasonable to suppose it might have been under selection for its contribution to the regulation of basal transcription. However, *GTB1* has also been predicted to interact with *ARGONAUTE* (*AGO*) proteins which function in the processing of small RNAs [66]. *AGO1* also shows evidence of a selective sweep in *A. arenosa* (Tables S3, S4), and *AGO4* also shows evidence of adaptive protein evolution (not shown). This however, may not be due to polyploidy *per se*, since *AGO* genes show evidence of selective sweeps in diploid species as well. For example, successive selective sweeps in an *Argonaute* gene in *Drosophila* species have been suggested to be associated with host-pathogen co-evolution [67].

The picture may be even more complex, since small RNAs have also been implicated in DNA double-strand break repair [68], [69], meiotic chromosome pairing [70], and mitotic and meiotic chromosome structure and segregation [71]–[73]. Indeed, AGOs have themselves been directly implicated in maintaining chromatin silencing during meiosis [71], [73]. These are fundamental genome maintenance processes strongly implicated in polyploid stabilization. Thus the true causes of selection on genes like *GTB1* or *AGO1* that are implicated in multiple distinct but interlocked processes provide extensive opportunities for follow-up studies to unravel the complexities of selection acting on interconnected pleiotropic genes, more than one of which may be under selection for different reasons.

For the chromosome synapsis gene *ASY1*, we confirmed differentiation among *A. arenosa* cytotypes of an amino acid substitution at a conserved position. *ASY1* is related to the *Hop1* gene in yeast, which plays important roles in the assembly of the synaptonemal complex and the regulation of homologous recombination [74]. In plants, these functions are conserved, e.g. [37], [75]. Synapsis is a process that has been hypothesized to play a role in meiotic stabilization of tetraploids [1], [4], and *ASY1* itself has been functionally implicated in polyploid meiosis. Expression of wheat *TaASY1* is affected by *Ph1*, and transgenic downregulation of *TaASY1* results in reduced synapsis but strengthened associations of homeologs at metaphase I [76]. If the derived *ASY1* allele in *A. arenosa* was important in polyploid evolution, as the signature of selection suggests, this implies that this gene may play a role in promoting meiotic stability in both allo- and autopolyploids. The presence of the derived *ASY1* allele at low frequency in the diploid gene pool suggests that standing variation for *ASY1*, rather than de novo mutation, may have been important for a rapid response to selection during tetraploid stabilization. This is consistent with findings in other species that genetic variation in diploids can affect meiotic stability after artificial genome doubling, e.g. [77].

Overall our data indicate that selection has acted on numerous genes in the tetraploid *A. arenosa* genome, providing specific candidate genes and mutations for mechanistic follow-up work. Some of this selection may have been on standing genetic variation in diploid *A. arenosa* that contributes to polyploid formation, for example by promoting unreduced (diploid) gamete formation. However, many of these selected alleles are likely to have been involved in the stabilization of fundamental biological processes after whole genome duplication. Our analysis implicates several fundamental processes and functions in adaptation to polyploidy, both supporting previous hypotheses about polyploid stabilization, such as modulation of meiosis, and suggesting new ones, such as involvement of a network associated with the regulation of core transcription. Finally, our analysis reveals an overlap of putatively selected genes and functions in *A. arenosa* with genes identified as essential in tetraploid yeast [12] and implicated in disease-associated failures of genome maintenance in humans. This suggests that key challenges faced by polyploids are shared across kingdoms and understanding how natural selection can circumvent these problems in a variety of species will provide important insights.

## Materials and Methods

### Plant material

Plants were grown directly from seeds collected from wild populations in the summers of 2009 and 2010. Seeds were collected in late June 2009 from the railway station in Triberg (TBG) in the Black Forest of southwestern Germany, and from a limestone outcrop near the Upfinger Steige (US), between Upfingen and Bad Urach in the Swabian Alb region of southwestern Germany. Seeds were collected in June 2010 from Kasparstein castle, in southern Austria (KA) and Berchtesgaden railway station (BGS) in southeastern Germany. Seeds were surface sterilized with 70% ethanol/0.05% Triton X-100, and then stratified at 4°C in the dark for six to eight days on 1/2×MS plates with 8% agar. Seeds were germinated in a tissue culture incubator at 16°C with 16 hour long days, and then transferred to soil (50% Sunshine Mix #4/50% fine vermiculite) and grown in a growth chamber with 16-hour long-day light cycles. Ploidy was verified by flow cytometry on at least one individual per population, and by testcrosses to known diploid and tetraploid individuals (the Streçno castle site in Slovakia, from which we also collected in 2010, was previously identified and confirmed as diploid by Luca Comai, UC Davis). Flow cytometry was also used to confirm that plants in our Streçno and Carpathians collections are diploid.

### Sequencing

Genomic DNA was extracted from one gram of leaf and inflorescence material from 6 to 10-week old plants using a DNeasy Maxi-Prep kit (Qiagen). We chose three individuals each from the TBG, US, BGS, and KA populations for sequencing. For all individuals, cluster generation and sequencing were performed using standard protocols provided with the kits used. Three of the genomic sequencing libraries were prepared using the Illumina Genomic Sample Preparation Kit for sequencing on the Illumina Genome Analyzer II (GAII). Each of the three individuals was sequenced on a single GAII lane for 85 sequencing cycles. The remaining nine libraries were prepared following the Illumina TruSeq Genomic Sample Preparation protocol for sequencing on the Illumina HiSeq 2000. For sequencing on the HiSeq, each sample was bar-coded and all nine samples were run across seven lanes for 100 sequencing cycles. Sequencing results in the form of FASTQ files were used as input for read mapping and analysis.

### Read mapping and error rate calculation

Short read mapping and processing was performed using SHORE version 5.0 [78]. Reads were mapped to the published *Arabidopsis lyrata* genome sequence using GenomeMapper, called by the SHORE subprogram mapflowcell (a list of all SHORE commands used during data processing are given in Text S2). Prior to mapping, we imposed a Sanger quality score cutoff of 30 for base calling. In addition, because errors can arise from both sequencing and read mapping, we assessed the full error rate by calculating the observed divergence between *A. arenosa* reads, the *A. lyrata* sequence mapped to, and the orthologous *A. thaliana* sequences following [79]. We selected a sample of 500,000 uniquely mapping reads from each individual, and produced local alignments of each read and the corresponding *A. lyrata* sequence [15] to the published *A. thaliana* TAIR 9 genome sequence using BLAST. For sequences that had a unique match to the *A. thaliana* reference (E-value cutoff = 1e^−5^), we then counted the number of changes between the *A. thaliana* sequence and the *A. lyrata* sequence or *A. arenosa* read, respectively. Because the number of observed changes reflects both evolutionary divergence and sequencing error, the excess number of changes on the *A. arenosa* reads relative to the *A. lyrata* sequence gives an estimate of error stemming from both sequencing and mapping (the contribution of sequencing error in the *A. lyrata* reference genome was assumed to be negligible). The estimated error rates were low, ranging from 0.1–0.2%.

### Genotyping

Consensus sequence outputs for each individual were produced using the SHORE consensus sub-program specifying the -v to write all intermediate data to a file. This allowed selection of only uniquely mapping reads exceeding the quality cutoff (see above), upon which all subsequent analysis was based. All downstream parsing of files was performed using custom PERL scripts (see Text S2).

To estimate the tetraploid genotype from each individual, we used a modification of the genotyping algorithm described in [80] modified to account for the three heterozygous states possible in a tetraploid (AAAa, AAaa, Aaaa), and also designed to call homozygotes (AAAA and aaaa) in the presence of sequencing errors. Given the uncertainty regarding the mode of inheritance and demographic history of *A. arenosa* populations, both of which can affect expected genotype frequencies, we estimated individual genotypes directly from the pileup of bases for each individual (see Text S1). Following [79], we defined the probability of the data D (the pileup of bases) given the genotype G for a given reference position
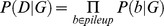
where b represents the state of a single mapped read at that position. Accounting for the three heterozygous states possible in a tetraploid, the probability of each base given the genotype was then defined as
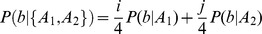
where *i* is the number of A_1_ alleles in the genotype considered, and *i*+*j* = 4. The probability of seeing a given allele was
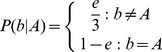
where *e* was the error rate measured for each individual, taking into account both mapping and sequencing error. The likelihood of each genotype given the base pileup was thus calculated, and the most probable genotype was accepted if its log-odds score was ≥2. Genotyping was only attempted for the subset of mapped sites where a) per-individual coverage was greater than 4× for all individuals, and b) no more than two variants were called among all individuals. Allele frequencies were then calculated from the inferred genotypes of all individuals. The performance of the genotyper in recapitulating genotype and allele frequencies was evaluated in simulations incorporating a stochastic sampling process similar to short read sequencing (see Text S1).

### Genomic analysis

All downstream data analysis made use of custom PERL and R scripts (see Text S2), in tandem with other software listed below. Summary statistics were generated using the libsequence evolutionary genetics software package [81]. Alignments of *A. arenosa* consensus sequences with *A. thaliana* and *A. lyrata* protein coding regions were generated using CLUSTALW 2.0. Alignments with <80% sequence identity among the three species were not included in further analysis.

All statistical tests were done using R version 2.11.0, and custom R scripts were written to perform genome wide analysis and tests for selection (see Text S2). Nucleotide diversity, π, is equivalent here to expected gametic heterozygosity estimated from inferred genotypes, where gametic heterozygosity equals the number of differences between any two sequences in a population sample [23]. Gametic heterozygosity was used for pairwise *F_ST_* and diversity summaries.

To test for selective sweeps, we first implemented a non-parametric test for atypical site frequency spectra (SFS) [27]. This test is particularly well suited to identifying regions with SFS skewed toward high frequency derived SNPs. We used both the *A. thaliana* and *A. lyrata* reference genomes to obtain the unfolded SFS for all SNPs in the *A. arenosa* data, assuming that sites identical in both outgroup sequences represent the ancestral state. To implement the test, we divided each of the genes in the dataset into 100 snp windows, and calculated the composite likelihood ratio (CLR) score for each window separately, and then identified outliers relative to the genome-wide distribution (Figure S4). To test for a local reduction in genetic diversity, we measured π/basepair and again identified outliers from the genome-wide pattern. We then made a list of the strongest candidates for selective sweeps by selecting the set of genes that fell both in the lowest 5% of the genome-wide distribution of π/basepair and also had one or more 100 bp windows that scored in the top 5% for CLR score. π and CLR were uncorrelated in our data (R^2^ = 0.014).

Gene interaction predictions were examined using the atPIN database [38] (http://bioinfo.esalq.usp.br/atpin/atpin.pl). We confirmed predicted interactions with literature searches and removed all that were based purely on phylogenetic relatedness of genes, but included all predicted as well as experimentally verified interactions with experimental support in *A. thaliana* or other species.

### Simulation analysis of mode of inheritance

We used coalescent simulations to generate neutral datasets using the software ms [82]. We used Watterson's estimator of 4N_e_u from the *A. arenosa* data to set realistic values of the population mutation rate. For each simulation, theta was set (using the -t switch) equal to the silent theta value from a randomly selected gene from the *A. arenosa* data (sampling with replacement). For all simulations, the sample size was set to 48 chromosomes (our *A. arenosa* sample size).

Disomic inheritance occurs when genetic diploidization effectively isolates homeologous chromosomes via consistent meiotic pairing preferences; this may happen immediately in allotetraploids, but in autotetraploids, pairing preferences may evolve much later. To model disomic inheritance, we simulated two sub-populations isolated for different lengths of time, and then drew two chromosomes from each per individual (representing the two homeologous chromosome pairs). We simulated data with the time since evolution of disomic inheritance (t_d_; in units of 4N generations) set over a range of values from t_d_ = 1 to t_d_ = 0.2, stepping by 0.2. For simulation of the evolution of disomic inheritance, the -I switch was used to simulate two sub-populations of sample size 24, and the -eN switch was used to specify the time in the past when the sub-populations split from the ancestral population – forward in time, this represented the isolation of homeologs (i.e. the evolution of disomic inheritance). Two chromosomes from each sub-population were then assigned to each simulated individual, representing the two pairs of homeologous chromosomes. We also simulated a fully tetrasomic set of chromosomes with no sub-division of chromosome pools. Simulated tetraploid genotypes were generated by randomly assigning four chromosomes to each of twelve “individuals”. The distribution of genotype frequencies for 12 “individuals” from fully tetrasomic simulations accurately recapitulated theoretical expectations for tetrasomic inheritance with bivalent pairing (Figure S3). For all models, we performed 500,000 simulation runs and pooled SNPs across all runs.

### PCR analysis of *ASY1*

We designed PCR primers to amplify a conserved region in the HORMA domain of *ASY*: 5′TTTGGTTTTCGTTTTGCTGA3′ and 5′GAGATTCAGCGTCCATAGGC3′. The high frequency SNP in this region causes a restriction site polymorphism for *XmnI*. Fragments were amplified from DNA from progeny of wild plants from five populations (Spisska, Slovakia; Carpathian Mountains, Tatras range, Slovakia; Gulsen, Austria; Koßelbach, Austria Berchtesgaden, Germany) using Taq polymerase (New England Biolabs) with an annealing temperature of 56°C. 10 µl of each product was digested with *XmnI* (New England Biolabs) and visualized on 1.5% agarose gels.

## Supporting Information

Figure S1 DNA content measured by flow cytometry. The y-axis is the number of cells counted in each sample, and the x-axis indicates flourescence intesntiy. The graph shows two example traces: in blue is a diploid *A. arenosa* genotype (from Strecno, Slovakia) and in red is the trace from a tetraploid (TBG). Flow cytometry was performed on leaf tissue and additional peaks at higher flourescence indicate endopolyploid cells. The first peak defines the ploidy, which lies at about 150 for diploids, and about 300 for tetraploids.
(EPS)

Figure S2 Average read coverage per individual. TBG1, TBG2 and US2 were sequenced on the GAII genome analyzer, while the rest were sequenced on the HiSeq platform.
(EPS)

Figure S3 Comparison of SFS from simulated and actual data from *A. arenosa* synonymous sites. (A) Expected neutral folded SFS from coalescent simulations under different inheritance models (1.0 = fully disomic; 0.2–0.8 intermediate with increasing time since diploidization; Auto = fully tetrasomic). (B) Comparison of *A. arenosa* polymorphism data to simulated tetrasomic (Auto), intermediate (t_d_ = 0.2) and disomic data (t_d_ = 1.0). (C) Inferred genotypes from simulated disomic, intermediate, tetrasomic (autotetraploid), and inferred *A. arenosa* genotypes from short read dataset.
(EPS)

Figure S4 CLR score histogram of *A. arenosa* gene regions. 5% cutoff is indicated with red dotted line.
(EPS)

Table S1 Fit of *A. arenosa* SNPs with simulated allo- and autotetraploid data. Comparison of our actual data with simulated data under distinct inheritance scenarios.
(DOCX)

Table S2 Summary of pairwise population differentiation. Shared variation, F_ST_ analysis and private polymorphism for the four *A. arenosa* populations included in our dataset.
(DOCX)

Table S3 High CLR/Low pi genes. List of 192 candidates for selective sweeps (genes ranked in the top 5% genomewide for CLR score, and in the bottom 5% for polymorphism (pi).
(PDF)

Table S4 Genes in selected overrepresented categories. Sweep candidates listed by over-represented functional category.
(DOCX)

Table S5 Signatures in annotated meiosis-related genes. CLR score and low pi signatures within annotated meiosis genes (TAIR 9).
(DOCX)

Table S6 Predicted interactions among candidate targets of selection. Interactions predicted by AtPIN database among genes included on our list of sweep candidates.
(DOCX)

Text S1 Detailed description of simulation analyses for genotypic inference.
(DOCX)

Text S2 List of commands used in data processing and analysis.
(DOCX)
