# Supplementary material for: Genetic Adaptation Associated with Genome-Doubling in Autotetraploid Arabidopsis arenosa
Source: PLoS Genet. 2012 Dec 20;8(12):e1003093. doi: 10.1371/journal.pgen.1003093 (PMC3527224; doi:10.1371/journal.pgen.1003093)
Supplement: Table S1 — Fit of A. arenosa SNPs with simulated allo- and autotetraploid data. Comparison of our actual data with simulated data under distinct inheritance scenarios. (DOCX) [file pgen.1003093.s005.docx]

**Table S1: Fit of *A. arenosa* SNPs with simulated allo- and autotetraploid data**

|  | χ^2^ Di (t_d_ =1.0) | χ^2^ Di (t_d_ = 0.2) | χ^2^ Tetr |
| --- | --- | --- | --- |
| Simplex (AAAa) | 2155.57 *** | 63.46 * | 21.33 (ns) |
| Duplex (AAaa) | 61.26 * | 27.31 (ns) | 10.49 (ns) |
| Triplex (Aaaa) | 12891.03 *** | 666.12 *** | 21.62 (ns) |
| Homozygote (aaaa) | 8166.50 *** | 178.28 *** | 37.87 (ns) |

Table S1 notes: χ^2^ values from comparing our actual data with genotype ratios in simulated datasets assuming complete and long-term disomic (allotetraploid) inheritance (Di; t_d_ =1.0), partial or recent allotetraploid inheritance (t_d_ = 0.2), or fully tetrasomic inheritance (Tetr); Significance: (ns) = not significant, * = *p*<0.05, ** = *p*<0.01, *** = *p*<0.001; χ^2^ test, 46 df.
