# Supplementary material for: Genetic Adaptation Associated with Genome-Doubling in Autotetraploid Arabidopsis arenosa
Source: PLoS Genet. 2012 Dec 20;8(12):e1003093. doi: 10.1371/journal.pgen.1003093 (PMC3527224; doi:10.1371/journal.pgen.1003093)
Supplement: Table S2 — Summary of pairwise population differentiation. Shared variation, FST analysis and private polymorphism for the four A. arenosa populations included in our dataset. (DOCX) [file pgen.1003093.s006.docx]

**Table S2: Summary of pairwise population differentiation**

| **Population Statistics for Synonymous Sites** | | |
| --- | --- | --- |
| **Comparison** | **Shared Var** | **F_ST_** |
| TBG vs. BGS | 0.483 | 0.063 |
| TBG vs. US | 0.487 | 0.060 |
| TBG vs. KA | 0.507 | 0.058 |
| BGS vs. US | 0.572 | 0.047 |
| BGS vs. KA | 0.531 | 0.058 |
| US vs. KA | 0.523 | 0.054 |
| *Average* | 0.518 | 0.057 |
|  |  |  |
| **Private Polymorphisms** | |  |
| TBG | 0.194 |  |
| BGS | 0.117 |  |
| US | 0.141 |  |
| KA | 0.123 |  |
| *Average* | 0.144 |  |
