# Supplementary material for: Genetic Adaptation Associated with Genome-Doubling in Autotetraploid Arabidopsis arenosa
Source: PLoS Genet. 2012 Dec 20;8(12):e1003093. doi: 10.1371/journal.pgen.1003093 (PMC3527224; doi:10.1371/journal.pgen.1003093)
Supplement: Table S3 — High CLR/Low pi genes. List of 192 candidates for selective sweeps (genes ranked in the top 5% genomewide for CLR score, and in the bottom 5% for polymorphism (pi). (PDF) [file pgen.1003093.s007.pdf]

Table S3: Genes scoring both in the top 5% High CLR and lowest 5% polymorphism (pi/site)

Gene ID	FD	Name	Description (TAIR9)	Locus	C	start	end	CLR	pi
AT1G02080	YES		transcriptional regulator	scaffold_100023.1	1	58885	72152	53.9	0.006
AT1G03060		SPIRRIG	WD-40, ARP2/3 complex	fgenesh1_pm.C_scaffold_1000187	1	860953	874764	46. 7	0.006
AT1G03370	YES		C2 calcium/lipid-binding	scaffold_100286.1	1	991664	999458	59. 6	0.009
AT1G04120	YES	ATMRP5	Inos. hexakisphosphate trans	scaffold_100368.1	1	1295351	1300899	41.5	0.008
AT1G04200	YES		unknown protein	fgenesh2_kg.1__350__AT1G04200.1	1	1343815	1348096	55.9	0.009
AT1G05160	YES	CYP88A3	CYTOCHROME P450	fgenesh2_kg.1__460__AT1G05160.1	1	1783564	1785765	51.6	0.006
AT1G05910	YES		CDC48-related	fgenesh2_kg.1__560__AT1G05910.1	1	2134991	2140813	51.8	0.007
AT1G06410		ATTPS7	trehalose-phosphatase	fgenesh2_kg.1__614__AT1G06410.1	1	2321113	2323840	41.4	0.007
AT1G06490		CalS7	callose synthase	fgenesh2_kg.1__623__AT1G06490.1	1	2347291	2357835	61.7	0.009
AT1G06710			Tetratricopeptide repeat	fgenesh1_pm.C_scaffold_1000521	1	2427851	2430817	42.9	0.009
AT1G06720			P-loop cont NTP hydrolase	fgenesh2_kg.1__649__AT1G06720.1	1	2431303	2437628	44.7	0.010
AT1G06970		CHX14	sodium:hydrogen antiporter	fgenesh1_pm.C_scaffold_1000542	1	2520146	2522884	50.1	0.010
AT1G07990	YES		SIT4 phosphatase-associated	scaffold_100816.1	1	2962192	2968069	56.8	0.009
AT1G08060	YES	MOM	RNA-dir chromatin silencing	fgenesh2_kg.1__815__AT1G08060.1	1	2990769	2999597	66.2	0.007
AT1G08140	YES	ATCHX6A	CATION/H+ EXCHANGER 6A	fgenesh2_kg.1__826__AT1G08140.1	1	3044708	3047585	44.5	0.007
AT1G08260		TILTED1	Sim POL2A, epsilon subunit	scaffold_100851.1	1	3094520	3110355	49.3	0.010
AT1G08600		ATRX	nucleic acid binding	fgenesh2_kg.1__878__AT1G08600.1	1	3245847	3254600	125.6	0.010
AT1G09570	YES	PHYA	red/far-red photoreceptor	fgenesh2_kg.1__1007__AT1G09570.1	1	3646701	3650422	43.8	0.007
AT1G10010	YES	AAP8	amino acid transporter	fgenesh2_kg.1__1066__AT1G10010.1	1	3858665	3861532	51.1	0.005
AT1G13980		GNOM	GTP:GDP antiporter.	fgenesh2_kg.1__1513__AT1G13980.1	1	5871288	5876063	53.8	0.006
AT1G15780	YES		unknown protein	fgenesh2_kg.1__1730__AT1G15780.1	1	6668884	6674406	52.3	0.009
AT1G16190			DNA repair protein RAD23,	fgenesh1_pm.C_scaffold_1001379	1	6805425	6808094	41.4	0.009
AT1G16445	YES		Putative rRNA methylase	fgenesh2_kg.1__1795__AT1G16445.1	1	6889332	6890695	40.3	0.010
AT1G16760			protein kinase family protein	fgenesh1_pm.C_scaffold_1001429	1	7018136	7021209	48.6	0.007
AT1G17220	YES	FUG1	translation initiation factor	fgenesh2_kg.1__1879__AT1G17220.1	1	7202110	7206862	41.6	0.010
AT1G17750	YES	PEPR2	LRR transmembrane kinase	fgenesh2_kg.1__1941__AT1G17750.1	1	7460306	7463646	44.5	0.009

AT1G17940			Endosomal targeting	scaffold_101999.1	1	7528732	7531153	55.6	0.010
AT1G18610	YES		Galactose oxidase/kelch repeat	Al_scaffold_0001_1930	1	7812179	7814774	41.9	0.007
AT1G20780		SAUL1	ubiquitin-protein ligase	scaffold_102325.1	1	8902926	8905750	48.7	0.009
AT1G20970	YES		Proton pump	fgenesh2_kg.1__2291__AT1G20970.1	1	9017774	9023199	44.4	0.010
AT1G26130			ATPase E1-E2 type family	scaffold_102929.1	1	11464602	11469235	55.5	0.008
AT1G27320		AHK3	cytokinin receptor	fgenesh2_kg.1__2882__AT1G27320.1	1	11958127	11962460	60.4	0.010
AT1G28420	YES	HB-1	transcription factor	fgenesh2_kg.1__3015__AT1G28420.1	1	12586954	12594490	64.0	0.008
AT1G30970	YES	SUF4	H3 methyltransferase recr	fgenesh2_kg.1__3297__AT1G30970.1	1	14100978	14104090	46.0	0.008
AT1G32750		HAF01	histone acetyltransferase	fgenesh1_pm.C_scaffold_1002616	1	15074521	15084457	52.5	0.010
AT1G44910		PRP40A	Binds CTD of NRPB1	scaffold_104215.1	1	22922841	22929936	61.2	0.007
AT1G48410	YES	AGO1	miRNA / siRNA binding	fgenesh2_kg.1__3931__AT1G48410.2	1	24571686	24577383	48.8	0.006
AT1G50030		TOR	regulates cell growth	fgenesh2_kg.1__4094__AT1G50030.1	1	26016993	26034266	113.0	0.005
AT1G64550		ATGCN3	stress-associated translation	fgenesh2_kg.2__21__AT1G64550.1	2	127056	131623	53.9	0.010
AT1G64570	YES	DUO3	Male gametophyte	fgenesh1_pg.C_scaffold_2000018	2	114642	119601	41.0	0.008
AT1G65440	YES	GTB1	txn elong, sim to yeast Spt6	scaffold_201171.1	2	9431705	9439106	44.6	0.004
AT1G66680	YES	AR401	Methyltransferase	fgenesh2_kg.2__894__AT1G66680.1	2	11599787	11602401	45.7	0.006
AT1G67370	YES	ASY1	DNA binding; Synapsis	scaffold_201546.1	2	12427384	12431660	41.6	0.007
AT1G68990	YES	MGP3	Phage type RNA polymerase	fgenesh2_kg.2__1158__AT1G68990.1	2	13508658	13513893	77.0	0.008
AT1G72390			unknown protein	fgenesh1_pm.C_scaffold_2001237	2	15369674	15375358	42.0	0.006
AT1G73100		SUVH3	histone methyltransferase	scaffold_202240.1	2	15697317	15699326	58.3	0.010
AT1G75120	YES	RRA1	Arabinose	fgenesh2_kg.2__1781__AT1G75120.1	2	16555529	16557187	40.3	0.009
AT1G76390	YES		armadillo/beta-catenin repeat	fgenesh2_kg.2__1924__AT1G76390.2	2	17126535	17129135	57.6	0.005
AT1G77300	YES	EFS	histone methyltransferase	fgenesh2_kg.2__2024__AT1G77300.1	2	17575543	17584392	41.5	0.008
AT1G77600			binding	scaffold_202722.1	2	17706076	17715880	43.8	0.006
AT1G79000		HAC1	H3,4 histone acetyltransferase	fgenesh2_kg.2__2199__AT1G79000.1	2	18450345	18457405	78.6	0.008
AT2G01970			endomembrane protein 70	scaffold_500119.1	5	599747	602432	49.0	0.006
AT2G03150	YES	emb1579	binding / calcium ion binding	fgenesh2_kg.5__215__AT2G03150.1	5	1622175	1629317	42.4	0.009
AT2G07360			SH3 domain-containing	fgenesh2_kg.3__2937__AT2G07360.1	3	15102025	15111256	53.1	0.009

AT2G21470	YES	SAE2	subunit SUMO activ enzyme	fgenesh2_kg.4__72__AT2G21470.2	4	335690	339164	48.5	0.010
AT2G27170		SMC3	cohesin complex	fgenesh1_pg.C_scaffold_4000806	4	11123922	11131646	50.3	0.009
AT2G28180	YES	ATCHX8	sodium:hydrogen antiporter	fgenesh2_kg.4__751__AT2G28180.1	4	11996877	11999773	51.3	0.009
AT2G30110		ATUBA1	ubiquitin-activating enzyme	fgenesh2_kg.4__981__AT2G30110.1	4	13972335	13976742	49.0	0.010
AT2G30800	YES	HVT1	helicase/ nucleic acid binding	scaffold_401579.1	4	14422891	14429036	43.3	0.007
AT2G31660		SAD2	nuclear transport	fgenesh2_kg.4__1151__AT2G31660.1	4	14941079	14948367	42.7	0.009
AT2G31910		ATCHX21	sodium:hydrogen antiporter	Al_scaffold_0004_1647	4	15058759	15061072	71.5	0.010
AT2G33100	YES	ATCSLD1	cellulose synthase	fgenesh2_kg.4__1307__AT2G33100.1	4	15676135	15679683	69.1	0.006
AT2G33360			unknown protein	scaffold_401909.1	4	15825206	15827167	62.6	0.008
AT2G34780	YES	MEE22	Endoreduplication, meristem	fgenesh2_kg.4__1501__AT2G34780.1	4	16518327	16523440	52.7	0.009
AT2G35110		GNARLED	Component of WAVE complex	scaffold_402107.1	4	16669221	16676921	62.4	0.007
AT2G36810	YES		ARM repeat superfamily	fgenesh2_kg.4__1721__AT2G36810.1	4	17480821	17492346	62.2	0.008
AT2G41960			unknown protein	fgenesh1_pm.C_scaffold_4001938	4	20532643	20537349	68.4	0.006
AT2G42030	YES		zinc finger family	fgenesh2_kg.4__2349__AT2G42030.1	4	20558546	20559784	49.8	0.008
AT2G42640	YES		serine/threonine kinase	scaffold_403137.1	4	20862931	20868040	43.9	0.007
AT2G45540	YES		WD-40 repeat family protein	fgenesh2_kg.4__2751__AT2G45540.1	4	22164127	22179045	66.2	0.005
AT2G45910			protein kinase family protein	fgenesh2_kg.4__2791__AT2G45910.1	4	22333945	22337638	62.8	0.007
AT2G46020	YES	AtBRM	SWI/SNF chromatin ATPase	fgenesh2_kg.4__2806__AT2G46020.1	4	22367249	22375636	52.8	0.005
AT2G46560			transducin / WD-40 repeat	scaffold_403625.1	4	22610606	22621478	53.1	0.007
AT3G01320	YES	SNL1	represses transcription	fgenesh2_kg.3__82__AT3G01320.1	3	312229	318718	55.8	0.008
AT3G02060	YES		DEAD/DEAH box helicase,	fgenesh2_kg.3__6__AT3G02060.1	3	6883	10827	50.9	0.005
AT3G02260		ASA1	affects CAB genes in darkness	Al_scaffold_0003_149	3	525686	543069	43.7	0.005
AT3G02490	YES		pentatricopeptide (PPR)	scaffold_300189.1	3	619503	621506	63.5	0.007
AT3G03050	YES	CSLD3	cellulose synthase	fgenesh2_kg.3__252__AT3G03050.1	3	863184	866941	40.7	0.008
AT3G03110		XPO1B	Exportin, nuclear transport	scaffold_300274.1	3	923030	930807	91.7	0.010
AT3G03710		RIF10	chloroplast, resp to P starv	scaffold_300355.1	3	1188069	1193604	65.1	0.009
AT3G04260	YES	PTAC3	DNA binding	fgenesh2_kg.3__387__AT3G04260.1	3	1434086	1438368	52.9	0.009
AT3G04740	YES	SWP	Mediator complex component	fgenesh2_kg.3__444__AT3G04740.1	3	1642370	1649101	44.0	0.010

AT3G05680		EMB2016	embryo defective 2016	scaffold_300625.1	3	2171106	2181370	44.6	0.004
AT3G06190	YES	BPM2	Stress response	scaffold_300699.1	3	2466231	2468185	46.4	0.007
AT3G06510		SFR2	beta-glucosidase	fgenesh2_kg.3__657__AT3G06510.1	3	2699390	2702553	42.3	0.008
AT3G06520	YES		agenet domain-containing	fgenesh1_pg.C_scaffold_3000604	3	2703257	2704877	42.4	0.010
AT3G08960			ARM repeat	fgenesh1_pm.C_scaffold_3000716	3	3541299	3547808	51.3	0.009
AT3G12270	YES	PRMT3	arginine methyltransferase 3	fgenesh1_pm.C_scaffold_3001021	3	4946247	4948805	48.3	0.008
AT3G12280	YES	RBR1	RETINOBLASTOMA-RELATED	fgenesh2_kg.3__1291__AT3G12280.1	3	4949348	4954125	45.6	0.010
AT3G12690		AGC1.5	serine/threonine kinase	fgenesh2_kg.3__1330__AT3G12690.1	3	5074506	5076313	54.6	0.004
AT3G12980		HAC5	H3/H4 acetyltransferase	scaffold_301479.1	3	5207622	5215128	42.0	0.007
AT3G15880		TPR4	WUS-interacting protein 2	fgenesh2_kg.3__1744__AT3G15880.2	3	6666381	6674783	40.9	0.010
AT3G15980			coatomer protein complex	fgenesh2_kg.3__1753__AT3G15980.3	3	6705091	6711398	40.6	0.010
AT3G16830	YES	TPR2	TPR2 (TOPLESS-RELATED 2)	fgenesh2_kg.3__1850__AT3G16830.1	3	7169993	7175817	80.7	0.006
AT3G17850			protein kinase, putative	Al_scaffold_0003_1880	3	7622254	7628626	59.1	0.008
AT3G19940			sugar transporter, putative	fgenesh1_pm.C_scaffold_3001761	3	8668093	8669841	50.6	0.008
AT3G21290			dentin sialophosphoprotein	fgenesh2_kg.3__2349__AT3G21290.1	3	9426110	9432161	58.1	0.009
AT3G42640		AHA8	H(+)-ATPase	fgenesh1_pm.C_scaffold_5000607	5	9482047	9485852	82.2	0.009
AT3G43300	YES	ATMIN7	ADP ribosylation, vesicle	Al_scaffold_0005_1053	5	10087736	10098667	73.4	0.005
AT3G50690	YES		leucine-rich repeat family	fgenesh2_kg.5__1409__AT3G50690.1	5	15263040	15264948	57.5	0.006
AT3G51120	YES		DNA binding	scaffold_502165.1	5	15525766	15531748	59.0	0.006
AT3G52020	YES	scpl39	serine-type carboxypeptidase	Al_scaffold_0005_2071	5	15921667	15923405	50.2	0.008
AT3G56320		OAS1	nucleotidyltransferase activity	Al_scaffold_0005_2541	5	17892029	17894465	50.2	0.009
AT3G57300		INO80	SWI/SNF ATPase family. HR.	scaffold_502896.1	5	18328418	18336179	60.9	0.006
AT3G58050	YES		unknown protein	fgenesh2_kg.5__2249__AT3G58050.1	5	18693419	18697895	48.0	0.006
AT3G63070			Tudor/PWWP/MBT domain	fgenesh2_kg.5__2803__AT3G63070.1	5	21032243	21039052	41.4	0.009
AT3G63130		RanGAP1	nuclear import	scaffold_503599.1	5	21055739	21057334	41.9	0.010
AT3G63180	YES	TKL	TIC-like (TKL); circadian	fgenesh2_kg.5__2812__AT3G63180.1	5	21068035	21072018	46.6	0.010
AT3G63460	YES		transducin family / WD-40	scaffold_503637.1	5	21184823	21191093	66.3	0.003
AT3G63480	YES		chromosome segregation	scaffold_503639.1	5	21194763	21197445	43.1	0.005

AT3G63510	YES		tRNA dihydrouridine synthase	fgenesh2_kg.5__2856__AT3G63510.1	5	21204115	21206243	44.7	0.004
AT3G63520	YES	CCD1		fgenesh2_kg.5__2858__AT3G63520.1	5	21206502	21209465	45.1	0.006
AT3G63530	YES	BB	ubiquitin-protein ligase	fgenesh1_pm.C_scaffold_5002321	5	21210071	21211424	42.3	0.005
AT4G00450	YES	CCT/CRP	homolog of mediator 12	Al_scaffold_0006_3973	6	24815697	24823918	51.5	0.009
AT4G03550	YES	ATGSL05	1,3-beta-glucan synthase	fgenesh2_kg.6__3348__AT4G03550.1	6	23049584	23055241	89.4	0.003
AT4G04920		SFR6	CBF pathway	fgenesh2_kg.6__3260__AT4G04920.1	6	22615236	22622080	88.5	0.007
AT4G08510	YES		unknown protein	fgenesh2_kg.6__2890__AT4G08510.1	6	18613515	18615571	42.8	0.008
AT4G11800			serine/threonine phosphatase	Al_scaffold_0006_3373	6	21532992	21537808	50.8	0.006
AT4G13750		NO VEIN	auxin-dependent development	Al_scaffold_0007_2977	7	14674453	14683067	40.5	0.009
AT4G19960	YES		K transmembrane transporter	fgenesh2_kg.7__2312__AT4G19960.1	7	9870116	9873440	48.9	0.009
AT4G24200			TFIIS family	fgenesh2_kg.7__1873__AT4G24200.1	7	7703110	7706133	41.7	0.008
AT4G24560	YES	UBP16	ubiquitin-specific protease	fgenesh2_kg.7__1819__AT4G24560.1	7	7538233	7543169	60.8	0.010
AT4G25920	YES		unknown protein	fgenesh2_kg.7__1658__AT4G25920.1	7	6912354	6913749	54.5	0.009
AT4G27000		RBP45C	RNA recognition motif, RNP-1	scaffold_701590.1	7	6428501	6431317	41.5	0.009
AT4G28470	YES	ATRPN1B	26S Proteasome reg sub S2 1B	scaffold_701413.1	7	5756684	5761963	60.3	0.006
AT4G29790			unknown protein	fgenesh2_kg.7__1214__AT4G29790.1	7	5123336	5129295	53.5	0.008
AT4G30150	YES		pre-rRNA processing	scaffold_701210.1	7	4943245	4950334	52.4	0.009
AT4G30790			autophagy	fgenesh2_kg.7__1108__AT4G30790.1	7	4617548	4621861	49.1	0.005
AT4G32620			polycomb transcription factor	fgenesh1_pm.C_scaffold_7000698	7	3730912	3736212	63.4	0.004
AT4G32820			TPR-like superfamily protein	scaffold_700897.1	7	3605838	3615607	45.9	0.007
AT4G33240			Vacuole organization	fgenesh2_kg.7__794__AT4G33240.2	7	3323061	3331049	51.6	0.009
AT4G34060	YES	DML3	demethylates CpG and CpHpG	fgenesh1_pg.C_scaffold_7000597	7	2967413	2973079	43.5	0.010
AT4G34370	YES	ARI1	E3 ubiquitin ligase	fgenesh1_pm.C_scaffold_7000531	7	2817345	2819871	40.6	0.010
AT4G35800	YES	NRPB1	RNA POLYMERASE II SUBUNIT	fgenesh2_kg.7__493__AT4G35800.1	7	2174483	2181369	43.0	0.008
AT4G35830		ACO	aconitase; TCA cycle	scaffold_700524.1	7	2164179	2169203	43.4	0.008
AT4G35890	YES		La domain-containing protein	fgenesh1_pg.C_scaffold_7000413	7	2110078	2112873	40.2	0.007
AT4G36080	YES		FAT domain-containing	fgenesh2_kg.7__457__AT4G36080.1	7	2021734	2039236	49.7	0.005
AT4G38350			hedgehog receptor	fgenesh2_kg.7__28__AT4G38350.1	7	72848	80899	49.0	0.007

AT4G38600		KAKTUS	HECT ubiquitin ligase	scaffold_700052.1	7	162169	170106	40.4	0.007
AT4G38780	YES		Pre-mRNA-processing-splicing	fgenesh2_kg.7__75__AT4G38780.1	7	225214	234801	60.2	0.007
AT4G39110	YES		Malectin/RLK	scaffold_700110.1	7	419084	421732	51.3	0.005
AT4G39450			unknown protein	fgenesh2_kg.7__156__AT4G39450.1	7	565783	573267	81.2	0.004
AT5G01770		RAPTOR2	partner of TOR kinase	fgenesh2_kg.6__23__AT5G01770.1	6	95244	102555	40.3	0.008
AT5G01990			auxin efflux carrier	fgenesh2_kg.6__4__AT5G01990.1	6	16498	18776	42.4	0.008
AT5G02310	YES	PRT6	ubiquitin-protein ligase;	fgenesh2_kg.6__143__AT5G02310.1	6	574878	583148	62.8	0.005
AT5G02880		UPL4	HECT ubiquitin-protein ligase	fgenesh2_kg.6__201__AT5G02880.1	6	781080	787337	44.3	0.009
AT5G03280		EIN2	ETHYLENE transporter	fgenesh2_kg.6__233__AT5G03280.1	6	928319	932810	43.9	0.008
AT5G04940		SUVH1	histone methyltransferase	fgenesh2_kg.6__415__AT5G04940.2	6	1715447	1717423	50.0	0.006
AT5G05470	YES	EIF2a	translation initiation factor	scaffold_600517.1	6	1997274	1998926	65.7	0.005
AT5G06110			DNAJ heat shock / cell division	scaffold_600585.1	6	2255114	2257340	42.3	0.008
AT5G07130		LAC13	Laccase; lignin catabolism	fgenesh1_pm.C_scaffold_6000548	6	2720833	2722806	40.7	0.010
AT5G07660	YES	SMC6a	component of SMC5/6,	fgenesh2_kg.6__735__AT5G07660.1	6	2977450	2984669	56.7	0.009
AT5G08520			myb family transcription factor	fgenesh2_kg.6__825__AT5G08520.1	6	3538503	3540808	87.6	0.009
AT5G08560	YES	ILP1	endoploidy represses Cyc2A	fgenesh2_kg.6__831__AT5G08560.1	6	3560720	3563393	70.3	0.005
AT5G08630			Flavonol synthase	scaffold_600900.1	6	3591406	3594930	45.5	0.008
AT5G11430			SPOC domain / TFS-II	fgenesh1_pg.C_scaffold_6000931	6	4565125	4568893	74.2	0.009
AT5G11470	YES		bromo-adjacent homology	fgenesh2_kg.6__1117__AT5G11470.1	6	4581419	4585153	60.0	0.007
AT5G12120		MXC9.8	translation elongation EF1B	fgenesh2_kg.6__1189__AT5G12120.1	6	4919268	4921103	54.7	0.008
AT5G12380		ANNEX8	Ca-dep phospholipid binding	fgenesh2_kg.6__1220__AT5G12380.1	6	5026984	5028448	54.9	0.009
AT5G13010		EMB3011	RNA helicase	fgenesh2_kg.6__1251__AT5G13010.1	6	5208775	5214720	47.6	0.008
AT5G14720	YES		protein kinase family protein	fgenesh2_kg.6__1447__AT5G14720.1	6	5948360	5952800	41.8	0.008
AT5G15540	YES	SCC2	sister-chromatid cohesion	scaffold_601585.1	6	6327475	6337206	40.3	0.010
AT5G15580	YES	LNG1	cell expansion leaf-length	fgenesh2_kg.6__1536__AT5G15580.1	6	6354461	6357890	64.2	0.009
AT5G15680			binding	fgenesh2_kg.6__1545__AT5G15680.1	6	6392479	6402096	50.5	0.010
AT5G15920		SMC5	component of SMC5/6,	fgenesh1_pm.C_scaffold_6001261	6	6493470	6496797	46.8	0.008
AT5G16280	YES		unknown protein	fgenesh2_kg.6__1612__AT5G16280.1	6	6646193	6654038	54.0	0.007

AT5G18410	YES	KLK	actin cytoskeleton, nucleation	fgenesh2_kg.6__1850__AT5G18410.2	6	7640675	7647925	47.0	0.005
AT5G18420	YES		unknown protein	Al_scaffold_0006_1759	6	7648883	7652553	58.1	0.008
AT5G19280	YES	KAPP	kinase associated phosphatase	fgenesh2_kg.6__1945__AT5G19280.1	6	8145565	8150237	40.2	0.010
AT5G23110			zinc finger	fgenesh2_kg.6__2302__AT5G23110.1	6	9836652	9853903	59.3	0.008
AT5G23450		LCBK1	D-erythro-sphingosine kinase/	fgenesh2_kg.6__2342__AT5G23450.2	6	10047267	10051160	41.4	0.010
AT5G25150	YES	TAF5	subunit of TFIID	fgenesh2_kg.6__2501__AT5G25150.1	6	11153543	11158319	46.9	0.010
AT5G27970	YES		ARM repeat superfamily	fgenesh1_pm.C_scaffold_6002211	6	13338190	13349179	53.5	0.010
AT5G42950			GYF domain; ABA response	fgenesh2_kg.8__540__AT5G42950.1	8	5398963	5405714	47.6	0.010
AT5G47490	YES		unknown protein	fgenesh2_kg.8__39__AT5G47490.1	8	149223	155190	42.5	0.008
AT5G47690	YES		binding	fgenesh2_kg.8__17__AT5G47690.1	8	82129	91174	44.2	0.005
AT5G47720	YES		acetyl-CoA C-acyltransferase,	fgenesh2_kg.8__12__AT5G47720.2	8	71958	74297	42.4	0.006
AT5G47730		SEC14	polyphosphoinositide-binding,	scaffold_800014.1	8	69333	71339	49.5	0.007
AT5G47820		FRA1	microtubule motor, kinesin	fgenesh2_kg.8__639__AT5G47820.2	8	9785302	9791160	91.4	0.008
AT5G52530	YES		dentin sialophosphoprotein	scaffold_801703.1	8	15188711	15191432	48.9	0.009
AT5G53150	YES		DNAJ heat shock N-terminal	fgenesh2_kg.8__1202__AT5G53150.1	8	15522544	15524796	40.9	0.008
AT5G57110	YES	ACA8	calmodulin binding	Al_scaffold_0008_2162	8	17902699	17909794	54.0	0.009
AT5G57360	YES	ZTL	clock-associated PAS protein	scaffold_802310.1	8	18069630	18072393	56.9	0.010
AT5G60040	YES	NRPC1	subunit of RNA polymerase III	fgenesh2_kg.8__1958__AT5G60040.1	8	19372056	19381850	41.3	0.006
AT5G60170	YES		RNA binding	fgenesh1_pg.C_scaffold_8002022	8	19443745	19448196	70.3	0.006
AT5G60980		NTF2	nuclear transport factor family	fgenesh1_pm.C_scaffold_8001690	8	19830353	19832364	47.6	0.008
AT5G61960		AML1	mei2-like	scaffold_802845.1	8	20245044	20249585	46.1	0.008
AT5G62050	YES	OXA1	Organelle Protein assembly	fgenesh2_kg.8__2195__AT5G62050.1	8	20301595	20304314	40.3	0.009
AT5G62190	YES	PRH75	DEAD/H-box RNA helicase	fgenesh2_kg.8__2212__AT5G62190.1	8	20372507	20375837	42.6	0.006
AT5G62640		ELF5	flowering time regulation	fgenesh2_kg.8__2263__AT5G62640.2	8	20611337	20614184	77.0	0.010
AT5G63610	YES	HEN3	homolog of CTD-binding CDK8	scaffold_803053.1	8	21038231	21039640	53.1	0.010
AT5G64070		PI-4KB1	phosphatidylinositol biosynth	fgenesh2_kg.8__2418__AT5G64070.1	8	21285128	21291604	40.9	0.006

Table S3 notes: ATG ID = Arabidopsis homolog name (TAIR 9); FD = fixed difference present ("YES"), Locus = A. lyrata locus name; C = A. lyrata chromosome number; start = start position on *A. lyrata* scaffold; end = end position on *A. lyrata* scaffold; CLR = composite likelihood ratio score for SFS outliers, pi = nucleotide diversity.
