# Supplementary material for: Genetic Adaptation Associated with Genome-Doubling in Autotetraploid Arabidopsis arenosa
Source: PLoS Genet. 2012 Dec 20;8(12):e1003093. doi: 10.1371/journal.pgen.1003093 (PMC3527224; doi:10.1371/journal.pgen.1003093)
Supplement: Table S4 — Genes in selected overrepresented categories. Sweep candidates listed by over-represented functional category. (DOCX) [file pgen.1003093.s008.docx]

**Table S4. General functions of genes from sweep candidate list**

| **General function** | | **Identifier** | **Gene name** |
| --- | --- | --- | --- |
| **Regulation of transcription** | | | |
|  | *Polymerases / pol subunits* | At1g68990 | *Mitochondrial RPOT* |
|  |  | At4g35800 | *NRPB1 (Pol II large SU)* |
|  |  | At5g60040 | *NRPC1 (Pol III subunit)* |
|  | *Pol II CTD interactors* | At1g44910 | *NRPB1 CTD-interactor* |
|  |  | At1g65440 | *GTB1* |
|  |  | At5g63610 | *CDKE;1 / HEN3* |
|  | *Pol II transcription factors* | At4g24200 | *TFIIS family* |
|  |  | At5g11430 | *TFIIS family* |
|  |  | At5g25150 | *TAF5 (TFIID subunit)* |
|  | *Pol II mediator complex* | At3g04740 | *STRUWWELPETER* |
|  |  | At4g00450 | *CENTER CITY* |
| **Epigenetic regulation** | |  |  |
|  | *Chromatin remodeling* | At1g30970 | *SUF4* |
|  |  | At1g65440 | *GTB1 (Spt6 homolog)* |
|  |  | At2g46020 | *AtBRM (SWI/SNF)* |
|  |  | At3g01320 | *SIN3-LIKE1* |
|  | *Histone acetyltransferases* | At1g32750 | *HAF01* |
|  |  | At1g79000 | *HAC1* |
|  |  | At3g12980 | *HAC5* |
|  | *Histone methyltransferases* | At1g73100 | *SUVH3* |
|  |  | At1g77300 | *EFS* |
|  |  | At5g04940 | *SUVH1* |
|  | *DNA demethylation* | At4g34060 | *DEMETER-LIKE3* |
|  | *RNA-mediated silencing* | At1g08060 | *MORPHEUS MOLECULE* |
|  |  | At1g48410 | *ARGONAUTE1* |
| **DNA repair / Homologous recombination** | | | |
|  |  | At1g08600 | *ATRX* |
|  |  | At1g16190 | *Rad23-like* |
|  |  | At2g46020 | *AtBRM* |
|  |  | At3g57300 | *INO80* |
|  |  | At5g07660 | *SMC6a* |
|  | | At5g15920 | *SMC5* |
| **Meiosis** | | | |
|  | *Regulation of meiosis* | At5g61960 | *AML1 (mei-2 like)* |
|  | *Synapsis* | At1g67370 | *ASYNAPSIS1* |
|  | *Sister chromatid cohesion* | At5g15540 | *SCC2* |
|  |  | At2g27170 | *SMC3* |
|  |  | At5g15920 | *SMC5* |
|  |  | At5g07660 | *SMC6a* |
| **Nucleocytoplasmic transport** | |  |  |
|  |  | At2g31660 | *SAD2* |
|  |  | At3g03110 | *XPO1B* |
|  |  | At3g08960 | *Nuclear pore component* |
|  |  | At3g63130 | *RanGAP1* |
|  | | At5g60980 | *NTF2* |
| **Cell division, growth and morphogenesis** | | | |
|  | *Cell cycle / Cell division* | At1g05910 | *CDC48-related* |
|  |  | At1G07990 | *SIT4-associated* |
|  |  | At1g08260 | *TILTED1 (DNA Pol ε)* |
|  |  | At1g13980 | *GNOM* |
|  |  | At1g16190 | *Rad23-like* |
|  |  | At1g50030 | *TOR* |
|  |  | At3g12280 | *RBR1* |
|  |  | At5g01770 | *RAPTOR2* |
|  | *Cytoskeleton / cell shape* | At1g03060 | *SPIRRIG* |
|  |  | At2g35110 | *GNARLED* |
|  |  | At5g18410 | *KLUNKER/PIROGI* |
|  |  | At5g47820 | *FRA1* |
|  | *Endoreduplication* | At2g34780 | *MEE22* |
|  |  | At5g08560 | *ILP1* |
|  | *Cell elongation* | At5g15580 | *LONGIFOLIA* |
|  | *Polarized cell growth* | At3g12690 | *AGC1.5* |
|  |  | At5g47730 | *SEC14* |
|  |  | At5g64070 | *Pi-4KBeta1* |
| **Protein degradation / Ubiquitination** | | | |
|  | *Ubiquitin activating* | At2g30110 | *ATUBA1* |
|  | *Proteases* | At3g63460 | *Part of CUL4-complex* |
|  |  | At4g24560 | *UBP16* |
|  | *Proteasome* | At4g28470 | *ATRPN1B* |
|  | *Ubiquitin-ligases* | At1g20780 | *SAUL1* |
|  |  | At3g63530 | *BIG BROTHER* |
|  |  | At4g34370 | *ARIADNE* |
|  |  | At4g38600 | *KAKTUS* |
|  |  | At5g02130 | *PRT6* |
|  |  | At5g02880 | *UPL4* |

Table S4 notes: Identifier is *A. thaliana* genome (ATG) gene number from TAIR 9 ([www.arabidopsis.org](http://www.arabidopsis.org)). Gene name gives common name or concise gene description. Functions are derived from annotations and GO category assignments from TAIR 9.
