# Supplementary material for: Genetic Adaptation Associated with Genome-Doubling in Autotetraploid Arabidopsis arenosa
Source: PLoS Genet. 2012 Dec 20;8(12):e1003093. doi: 10.1371/journal.pgen.1003093 (PMC3527224; doi:10.1371/journal.pgen.1003093)
Supplement: Table S5 — Signatures in annotated meiosis-related genes. CLR score and low pi signatures within annotated meiosis genes (TAIR 9). (DOCX) [file pgen.1003093.s009.docx]

**Table S5: Signatures in annotated meiosis-related genes**

| **Gene ID** | **Gene name** | ***A. lyrata* locus** | **clr** | **pi** |
| --- | --- | --- | --- | --- |
| AT1G01690 | PRD3 | Al_scaffold_0001_57 | *** | - |
| AT1G04730 | CTF18 | scaffold_100442.1 | - | - |
| AT1G08880 | HTA5 | scaffold_100916.1 | - | - |
| AT1G10710 | PHS1 | Al_scaffold_0001_1062 | - | - |
| AT1G14750 | SDS | fgenesh2_kg.1__1616__AT1G14750.1 | - | - |
| AT1G22275 | ZYP1b | scaffold_102525.1 | - | - |
| AT1G34355 | PS1 | fgenesh2_kg.1__3543__AT1G34355.1 | - | - |
| AT1G54690 | HTA3 | scaffold_704114.1 | - | *** |
| AT1G63990 | ATSPO11-2 | fgenesh2_kg.2__87__AT1G63990.1 | - | - |
| AT1G66170 | MMD1 | scaffold_201325.1 | - | - |
| AT1G67370 | ASY1 | scaffold_201546.1 | *** | *** |
| AT1G77320 | MEI1 | scaffold_202694.1 | - | - |
| AT1G77390 | CYCA1 | fgenesh2_kg.2__2033__AT1G77390.1 | - | - |
| AT2G06510 | RPA1a | scaffold_303391.1 | - | - |
| AT2G21800 | EME1a | fgenesh2_kg.4__105__AT2G21800.1 | - | - |
| AT2G22140 | EME1b | fgenesh2_kg.4__138__AT2G22140.1 | - | - |
| AT2G27170 | SMC3 | fgenesh1_pg.C_scaffold_4000806 | *** | *** |
| AT2G31970 | RAD50 | fgenesh1_pg.C_scaffold_4001300 | *** | - |
| AT2G40550 | ETG1 | fgenesh2_kg.4__2172__AT2G40550.1 | - | - |
| AT2G47980 | SCC3 | scaffold_403776.1 | - | *** |
| AT3G02680 | NBS1 | fgenesh2_kg.3__209__AT3G02680.1 | - | - |
| AT3G02980 | MCC1 | fgenesh2_kg.3__245__AT3G02980.1 | - | - |
| AT3G13170 | ATSPO11-1 | Al_scaffold_0003_1340 | - | - |
| AT3G18524 | MSH2 | fgenesh2_kg.3__2059__AT3G18524.1 | - | - |
| AT3G20475 | MSH5 | fgenesh2_kg.3__2273__AT3G20475.1 | - | - |
| AT3G23890 | TOPII | scaffold_302902.1 | *** | *** |
| AT3G24495 | MSH7 | fgenesh2_kg.3__2659__AT3G24495.1 | - | - |
| AT3G25100 | CDC45 | fgenesh2_kg.3__2716__AT3G25100.1 | - | - |
| AT3G27730 | RCK | scaffold_500731.1 | - | - |
| AT3G48190 | ATM | fgenesh1_pm.C_scaffold_5000964 | *** | - |
| AT3G54670 | SMC1 | fgenesh1_pm.C_scaffold_5001543 | *** | - |
| AT3G57300 | INO80 | scaffold_502896.1 | *** | *** |
| AT3G59550 | SYN3 | fgenesh2_kg.5__2414__AT3G59550.1 | - | - |
| AT4G00020 | EDA20 | fgenesh2_kg.6__43__AT4G00020.1 | - | - |
| AT4G02070 | MSH6 | scaffold_604079.1 | - | - |
| AT4G09140 | MLH1 | scaffold_603241.1 | - | - |
| AT4G14180 | PRD1 | fgenesh1_pm.C_scaffold_7002324 | - | - |
| AT4G14220 | RHF1a | fgenesh2_kg.7__2921__AT4G14220.1 | - | - |
| AT4G17380 | MSH4 | fgenesh2_kg.7__2572__AT4G17380.1 | *** | - |
| AT4G21270 | ATK1 | scaffold_702296.1 | - | - |
| AT4G22970 | ESP | fgenesh2_kg.7__2002__AT4G22970.1 | *** | - |
| AT4G25540 | MSH3 | fgenesh2_kg.7__1706__AT4G25540.1 | *** | - |
| AT4G29170 | ATMND1 | fgenesh2_kg.7__1273__AT4G29170.1 | - | - |
| AT4G30870 | MUS81 | fgenesh2_kg.7__1096__AT4G30870.1 | - | - |
| AT4G31400 | CTF7 | fgenesh2_kg.7__1034__AT4G31400.1 | - | - |
| AT4G35520 | MLH3 | fgenesh1_pm.C_scaffold_7000426 | - | - |
| AT5G05490 | BP2 | fgenesh1_pm.C_scaffold_6000400 | - | - |
| AT5G07660 | SMC6A | fgenesh2_kg.6__735__AT5G07660.1 | *** | *** |
| AT5G15540 | SCC2 | scaffold_601585.1 | *** | *** |
| AT5G15920 | SMC5 | fgenesh1_pm.C_scaffold_6001261 | *** | *** |
| AT5G16270 | SCC1 | scaffold_601664.1 | - | - |
| AT5G40840 | SYN2 | fgenesh2_kg.7__3327__AT5G40840.1 | *** | - |
| AT5G48390 | ATZIP4 | fgenesh1_pm.C_scaffold_8000591 | - | - |
| AT5G48720 | XRI | fgenesh2_kg.8__753__AT5G48720.1 | - | - |
| AT5G51330 | DYAD | fgenesh2_kg.8__1020__AT5G51330.1 | - | - |
| AT5G54260 | MRE11 | fgenesh2_kg.8__1327__AT5G54260.1 | *** | - |
| AT5G61460 | SMC6B | fgenesh2_kg.8__2121__AT5G61460.1 | - | - |
| AT5G63540 | RMI1 | scaffold_803044.1 | - | - |
| AT5G63920 | TOP3a | fgenesh2_kg.8__2400__AT5G63920.1 | *** | - |

**Table S5 notes:** Meiosis-related genes (as annotated in TAIR 9); *** indicates significance at α = 0.05 for outlier SFS (clr) or lowest 5% genome-wide for pi/site (pi).
