# Supplementary material for: Genetic Adaptation Associated with Genome-Doubling in Autotetraploid Arabidopsis arenosa
Source: PLoS Genet. 2012 Dec 20;8(12):e1003093. doi: 10.1371/journal.pgen.1003093 (PMC3527224; doi:10.1371/journal.pgen.1003093)
Supplement: Table S6 — Predicted interactions among candidate targets of selection. Interactions predicted by AtPIN database among genes included on our list of sweep candidates. (DOCX) [file pgen.1003093.s010.docx]

**Table S6: Predicted interactions among candidate targets of selection**

| **Gene ID** | **Gene Description (TAIR 9)** | **Known** | **Predicted int.** |
| --- | --- | --- | --- |
| AT2G35110 | GNARLED, actin nucleation. | 10 | AT5G18410 |
| AT5G18410 | KLUNKER; SCAR complex | 6 | AT2G35110 |
| AT1G27320 | AHK3; cytokinin receptor | 90 | AT5G13010 |
| AT5G13010 | EMB3011; RNA helicase | 61 | AT1G27320 |
| AT1G20970 | Proton pump | 32 | AT1G64550 |
| AT1G64550 | ATGCN3; transporter; | 45 | AT1G20970 |
| AT3G01320 | SIN3-like 1; SNL1 | 20 | AT4G32620 |
| AT4G32620 | Enhancer polycomb-like transcription | 2 | AT3G01320 |
| AT4G27000 | ATRBP45C; RNA binding | 12 | AT5G47720 |
| AT5G47720 | acetyl-CoA C-acyltransferase, putative | 64 | AT4G27000 |
| AT3G15880 | TOPLESS-RELATED 4 | 3 | AT3G16830 |
| AT3G16830 | TPR2 (TOPLESS-RELATED 2) | 2 | AT3G15880 |
| **TOR-network** | |  |  |
| AT1G50030 | TOR (TARGET OF RAPAMYCIN) | 48 | AT5G01770 AT3G19940 |
| AT3G12690 | AGC1.5; serine/threonine kinase | 93 | AT3G19940 |
| AT3G19940 | sugar transporter, putative | 121 | AT1G50030 AT3G12690 |
| AT5G01770 | RAPTOR2 | 100 | AT1G50030 |
| **UPL4 network** | |  |  |
| AT4G35830 | aconitate hydratase, cytoplasmic / aconitase |  | AT5G02880 |
| AT4G38600 | KAK (KAKTUS); ubiquitin-protein ligase | 23 | AT5G02880 |
| AT5G02880 | UPL4; ubiquitin-protein ligase; | 28 | AT4G35830 AT4G38600 |
| **Transcription Network** | |  |  |
| AT1G02080 | transcriptional regulator-related | 48 | AT1G32750 AT5G63610 |
| AT1G06410 | ATTPS7; trehalose-phosphatase | 8 | AT5G08630 |
| AT1G05910 | cell division cycle protein 48-related | 66 | AT5G08630 AT3G12280 |
| AT1G15780 | unknown protein | 65 | AT4G35800 AT5G63610 |
| AT1G16190 | DNA repair protein RAD23, putative; | 70 | AT4G35800 |
| AT1G32750 | HAF01; DNA binding / histone acetyltransferase | 53 | AT5G25150 AT3G57300 |
| AT1G44910 | Binds carboxyl-terminal domain (CTD) of NRPB1 | 32 | AT4G35800 AT3G03110 |
| AT1G65440 | GTB1; transcr, chromatin structure | 44 | AT4G35800 |
| AT1G73100 | SUVH3 (SU(VAR)3-9 HOMOLOG 3); histone methyltransferase | 2 | AT5G25150 AT5G04940 |
| AT2G27170 | SMC3, cohesin complex, viability and sister chromatid alignment. | 48 | AT3G03110 AT5G15540 |
| AT2G30110 | ATUBA1; ubiquitin-protein ligase | 36 | AT4G35800 |
| AT2G34780 | MEE22, endoreduplication, stem cells | 6 | AT3G12280 |
| AT3G03110 | XPO1B; member of the exportin family, nuclear transport | 74 | AT3G63130  AT2G27170 AT1G44910 |
| AT3G12280 | RBR1 (RETINOBLASTOMA-RELATED 1); transcription factor binding; | 285 | AT5G25150 AT5G47820 AT1G05910 AT2G34780 |
| AT3G12980 | HAC5; H3/H4 histone acetyltransferase | 36 | AT4G35800 AT5G63610 |
| AT3G57300 | INO80 (INO80 ORTHOLOG); DNA homologous recombination (HR). | 50 | AT1G32750 AT5G63610 |
| AT3G63130 | RANGAP1 | 37 | AT3G03110 |
| AT4G33240 | (PtdIns3P) 5-kinase | 53 | AT4G35800 |
| AT4G35800 | NRPB1 (RNA POLYMERASE II LARGE SUBUNIT); DNA binding / DNA-directed RNA polymerase; Encodes the unique largest subunit of nuclear DNA-dependent RNA polymerase II; the ortholog of budding yeast RPB1 and a homolog of the E. coli RNA polymerase beta prime subunit. | 144 | AT2G30110 AT1G65440 AT1G16190 AT3G12980 AT5G63610 AT1G15780 AT4G33240 AT1G44910  AT5G25150 |
| AT4G36080 | FAT domain-containing protein | 32 | AT5G25150 |
| AT5G08630 | DDT domain, Flavonol synthase | 30 | AT1G06410 |
| AT5G04940 | SUVH1; histone methyltransferase | 5 | AT1G73100 |
| AT5G15540 | ATSCC2; sister-chromatid cohesion, chromosome organization in meiosis | 16 | AT5G47690 AT2G27170 |
| AT5G25150 | TAF5 (TBP-ASSOCIATED FACTOR 5); subunit of the general transcription factor IID (TFIID). | 78 | AT1G32750 AT1G73100 AT3G12280 AT4G36080 |
| AT5G47690 | binding | 13 | AT5G15540 |
| AT5G47820 | FRA1 (FRAGILE FIBER 1); kinesin | 7 | AT3G12280 |
| AT5G63610 | HEN3/CDKE;1 (CYCLIN-DEPENDENT KINASE E;1); ATP binding / kinase/ protein kinase/ protein serine/threonine kinase | 83 | AT4G35800 AT3G57300 AT3G12980 AT1G02080 AT1G05910 AT1G15780 |

**Table S6 notes**: “Known” = total number of predicted interactions deposited in the atPIN database.
