# Supplementary material for: Genetic Adaptation Associated with Genome-Doubling in Autotetraploid Arabidopsis arenosa
Source: PLoS Genet. 2012 Dec 20;8(12):e1003093. doi: 10.1371/journal.pgen.1003093 (PMC3527224; doi:10.1371/journal.pgen.1003093)
Supplement: Text S1 — Detailed description of simulation analyses for genotypic inference. (DOCX) [file pgen.1003093.s011.docx]

**Text S1: Simulation analysis of genotype inference**

Genotyping algorithms depend on the representation of alleles among sequenced reads to calculate the most probable genotype, and are sensitive to random sampling of alleles during sequencing. Analysis of tetraploids may present a particular problem in this regard due to the presence of five different possible genotypes, so we explicitly tested the performance of our genotyper in recapitulating tetraploid genotypes by simulating the sampling of alleles during short read sequencing. We generated “true” tetraploid SNP genotypes and allele frequencies from coalescent simulations as described in the Materials and Methods. We simulated 200,000 runs under the tetrasomic model, and calculated the tetraploid genotypes for each of 12 simulated “individuals”. Next, we subjected the genotypes to a sampling and error generation process similar to what occurs in short read sequencing, using the following approach.

At each simulated SNP we simulated the sequencing coverage for each “individual” by sampling from a Poisson distribution with parameter equal to the average coverage of the corresponding individual in the *A. arenosa* data. To simulate the representation of alleles sequenced on the flowcell for each individual, we sampled from a binomial distribution with sample size equal to the simulated coverage and probability of success equal to the proportion of derived alleles in the tetraploid genotype (i.e. 0, 1/4, 1/2, 3/4, or 1). Here a “success” meant the derived allele was sequenced on that “read”. We thus obtained a vector of size equal to the simulated coverage made up of ones and zeros, where each one represented a read that sequenced the derived allele and each zero represented a read that sequenced the ancestral allele. To simulate sequencing error, we changed each element of the vector (each “read”) from one to zero (or vice versa) with probability e/3, where e was the error rate measured for the *A. arenosa* individual corresponding to the simulated individual. This approximated the sampling of alleles during short read sequencing, in the absence of a bias in allele representation prior to cluster generation. We then inferred the “sequenced” genotype of each individual using our genotyping algorithm (see Materials and Methods), based on the representation of the derived and ancestral alleles at the site for each individual. Finally, we compared the inferred genotypes after “sequencing” to the true genotypes from the coalescent simulations.

Overall, the genotyper recapitulated the “genome-wide” allele and genotype frequencies accurately, miscalling genotypes at a minority of sites ranging from 14% in the “individual” with the lowest coverage to 2% in the “individual” with the highest coverage. Additionally, and as expected, the agreement between the true and “sequenced” genotypes was a positive function of coverage within individuals. Importantly, the sequenced SFS was not significantly different from the tetrasomic SFS (Mann-Whitney U Test (MWU) *p*=0.9; Figure 1), but was significantly different from the t=0.2 disomic model (MWU *p*<0.03; Figure 1; see Materials and Methods). Additionally, average genotype frequencies as a function of allele frequencies were a much better fit between the true and sequenced genotypes for the tetrasomic model than between the sequenced tetrasomic genotypes and the disomic model (Table 1). Finally, we saw no large differences between true and “sequenced” genotypes at particular sites that might contribute to spurious inferences of selection and/or demography.

In general, this simulation analysis suggests that our genotyper is fairly robust to random allele sampling and error during sequencing and allows reliable inferences to be made from the data. This observation bears upon two of the major results from our analysis of the *A. arenosa* data: (1) the better fit of our data to the tetrasomic model than to any of the disomic models, and (2) the skewed SFS observed at a small number of loci in the *A. arenosa* dataset. The analysis presented here suggests that those results reflect true features of the population sample, and are not artifacts of our methodology.

**Table 1: the ratio of goodness-of-fit statistics for the “sequenced” genotype frequencies vs. the tetrasomic model to the sequenced genotype frequencies vs. disomic model**

| Genotype Category | GOF Ratio |
| --- | --- |
| Simplex | 6.063011 |
| Duplex | 5.201724 |
| Triplex | 15.96299 |
| Homozygous | 11.33924 |

**Figure 1:**

**Figure 1:** Site frequency spectra (SFS) of inferred “sequenced” tetrasomic data, and “true” tetrasomic and disomic data. Frequency is shown on the X axis and proportion of counts on the Y axis.
