# Supplementary material for: Genetic Adaptation Associated with Genome-Doubling in Autotetraploid Arabidopsis arenosa
Source: PLoS Genet. 2012 Dec 20;8(12):e1003093. doi: 10.1371/journal.pgen.1003093 (PMC3527224; doi:10.1371/journal.pgen.1003093)
Supplement: Text S2 — List of commands used in data processing and analysis. (DOCX) [file pgen.1003093.s012.docx]

**Supplemental Text S2**

This section gives the scripts used for analyses described in the paper.

**1. SHORE commands used:**

shore illumina2flat -v Fastq -e Shore -a genomic -i FLOWCELL_ID(INTEGER) -x PATH_TO_FASTQ_FILE -o PATH_TO_OUTPUT_FOLDER -Q illumina

shore mapflowcell -o PATH_TO_FLOWCELL_FOLDER -f PATH_TO_REFERENCE -n 10 -g 3 -c NUMBER_OF_CPU

shore merge -p LIST_ALL_FLOWCELL_FOLDERS_TO_MERGE -D PATH_TO_OUTPUT_FOLDER

shore consensus -r -v -n NAME -f PATH_TO_REFERENCE -o PATH_TO_OUTPUT_FOLDER -i PATH_TO_MAP.LIST_FOLDER

**2. Perl script for parsing shore output by gene/intergenic region**

#!/usr/bin/perl -w

$e=0;

$g = 0;

$lastloc = "Left";

$lastgene = 0;

$chrom = $ARGV[0];

$pathos = $ARGV[1];

$infile1 = "Araly1_GeneModels_FilteredModels6.gff";

&getgenes;

$gtotal=$g;

$file = "consensus_summary$chrom.txt";

$ntl = $gtotal-1;

$g=0;

$go=0;

$gstart = $genestart{$g};

$gend = $geneend{$g};

while($g<$gtotal) {

if($go==0) {

$h=$g+1;

$thispath = "$pathos/genefiles/$locus{$g}";

$gstart = $genestart{$g};

$gend = $geneend{$g};

$nextpath = "$pathos/genefiles/$locus{$h}";

system("mkdir $thispath");

system("mkdir $nextpath");

$nextgene=$genestart{$h};

$outfile3 = "$nextpath/Upstream.$locus{$h}.$file.out";

$outfile1 = "$thispath/$locus{$g}.$file.out";

$outfile2 = "$thispath/Downstream.$locus{$g}.$file.out";

$outfile0 = "$thispath/Upstream.$locus{$g}.$file.out";

$go=1;

}

&sites;

$g++;

$go=0;

#print "$g";

}

exit;

sub getgenes {

print "getting gene data....\n";

$begin = 1;

$go = 1;

$plus=1;

$g=0;

$e=0;

$cdssize{$g} =0;

open(INFILE,"$infile1");

while(<INFILE>) {

chomp($_);

@array = split('\t',$_);

$c{$g} = $array[0];

$c{$g} =~ s/scaffold_//;

if($c{$g}==$chrom) {

if($array[2] eq CDS) {

$name = pop(@array);

@nom = split('\;',$name);

$tag = $nom[0];

@quote = split('\"',$tag);

$prot = $nom[1];

@pro = split('\s+',$prot);

$id = $pro[2];

$ex = $nom[2];

if($go == 1) {

$thisid = $id;

$genestart{$g} = $array[3];

$go=0;

}

unless($thisid == $id) {

if($c{$g}>$chrom) {

last;

}

$genesize{$g}=$geneend{$g}-$genestart{$g};

$genesize{$g}++;

$alygenes++;

#$locus{$g}=$thisloc;

$ortholog{$g}=0;

$flanks{$g}=0;

$emax{$g}=$e;

#print "$g $locus{$g} $genestart{$g} $geneend{$g} $genesize{$g} $emax{$g} $compliment{$g}\n";

$g++;

$cdssize{$g} =0;

$e=0;

$thisid=$id;

$genestart{$g} = $array[3];

$first=pop(@nom);

}

if($array[6] eq '+') {

$compliment{$g}=0;

}

else {

$compliment{$g}=1;

}

$geneend{$g} = $array[4];

$locus{$g}=$quote[1];

$e++;

}

}

}

close(INFILE);

$gtotal=$g;

}

sub sites {

open(IN,"$pathos/$file");

while(<IN>) {

#print OUT "here!\n";

chomp($_);

@ln = split('\t',$_);

if($ln[1] > $lastgene && $ln[1] < $gstart) {

if($g==0) {

open(OUT,">>$outfile0");

print OUT "$_\n";

close(OUT);

}

}

if($ln[1] >= $gstart && $ln[1] <= $gend) {

@ln2 = @ln;

$ln2[1] = $ln2[1]-$gstart;

$ln2[1]++;

$line = join("\t",@ln2);

open(OUT,">>$outfile1");

print OUT "$line\n";

close(OUT);

}

if($ln[1] > $gend && $ln[1] < $nextgene) {

open(OUT,">>$outfile2");

print OUT "$_\n";

close(OUT);

if($g>0) {

open(OUT,">>$outfile0");

print OUT "$_\n";

close(OUT);

}

}

if($ln[1] == $nextgene-1) {

$lastgene = $geneend{$g};

$lastloc = $locus{$g};

$g++;

$thispath = "$pathos/genefiles/$locus{$g}";

$gstart = $genestart{$g};

$gend = $geneend{$g};

if($g<=$ntl) {

$h=$g+1;

$nextgene=$genestart{$h};

if($g>0 && $g<$ntl) {

$nextpath = "$pathos/genefiles/$locus{$h}";

system("mkdir $nextpath");

}

}

if($g==$ntl) {

$nextgene=1000000000;

}

$outfile0 = "$nextpath/Upstream.$locus{$h}.$file.out";

$outfile1 = "$thispath/$locus{$g}.$file.out";

$outfile2 = "$thispath/Downstream.$locus{$g}.$file.out";

}

}

}

**##Combining data across individuals**

chr<-1

n<-12

qcutoff<-30

f<-read.csv("AlyGenesOriented.out",header=F)

f<-subset(f,f$V2==chr)

path<-"/n/bomblies_lab2/consensusdata/"

bases<-c("A","C","G","T","-")

bcols<-6:10

prefix<-c("","Upstream.","Downstream.")

for(g in 1:length(g) {

locus<-as.vector(f$V1[g])

for(l in 1:3) {

locs<-0

for(i in 1:n) {

loc<-0

loc<-try(read.table(paste(path,"arenosa",i,"con/ConsensusAnalysis/supplementary_data/genefiles/",locus,"/",prefix[l],locus,".consensus_summary",chr,".txt.out",sep="")))

if(length(loc)>4) {

qwalz<-cbind(loc[,1:2],loc[,49],loc[,51],loc[,53],loc[,55])

loc<-cbind(loc[,1:2],loc[,45],loc[,25:31])

loc$V11<-factor("X",levels=c("C","A","G","T","-","X"))

for(k in 1:length(loc[,1])) {

counts<-as.vector(loc[k,bcols])

gtc<-as.matrix(counts[order(counts,decreasing=T)])

gt<-bases[order(counts,decreasing=T)]

qwal<-as.matrix(qwalz[k,3:6])

qwal<-qwal[order(counts[1:4],decreasing=T)]

loc[k,6]<-gtc[1]

loc[k,7]<-0

if(gtc[1]>0 & qwal[1]>=qcutoff) {

loc[k,4]<-gt[1]

if(gtc[2]>0 & qwal[2]>=qcutoff) {

loc[k,11]<-gt[2]

loc[k,7]<-gtc[2]

}

}

}

loc<-cbind(loc[,1:4],loc[,11],loc[,5:7])

if(i==1 & length(loc)>4) {

locs<-loc

}

if(i>1 & length(loc)>4) {

loc<-cbind(loc$V2,loc[,4:8])

locs<-merge(locs,loc,by.x="V2",by.y="loc$V2")

}

}

}

write.table(locs,paste("ArenosaSitesCombined/",prefix[l],locus,".sites.out",sep=""),quote=F,row.names=F,col.names=F)

}

}

**##Genotyper**

chr<-1

n<-12

f<-read.csv("AlyGenesOriented.out",header=F)

f<-subset(f,f$V2==chr)

temp<-numeric(4)

majs<-5*1:n

majs<-majs-1

majcs<-majs+3

mins<-majs+1

mincs<-majs+4

leels<-mins+1

prefix<-c("","Upstream.","Downstream.")

for(a in 1:length(f$V1)) {

locus<-as.vector(f$V1[a])

temp[1]=locus

for(l in 1:3) {

locs<-try(read.table(paste("ArenosaSitesCombined/",prefix[l],locus,".sites.out",sep="")))

if(length(locs)>4) {

locs[,leels]<-0

for(k in 1:length(locs[,1])) {

aa<-as.vector(locs[k,3])

for(i in 1:n) {

ta<-as.vector(locs[k,majs[i]])

if(ta==aa) {

B=locs[k,mincs[i]]

A=locs[k,majcs[i]]

}

if(ta!=aa) {

A=locs[k,mincs[i]]

B=locs[k,majcs[i]]

}

probs<-numeric(5)

for(g in 0:4) {

if(g==0) {

PA=(e[n]/3)^A

PB=(1-(e[n]/3))^B

}

if(g>0 & g<4) {

PA = ((g/4)*(1-(e[n]/3)) + (1-(g/4))*(e[n]/3))^A

PB = ((1-(g/4))*(1-(e[n]/3)) + (g/4)*(e[n]/3))^B

}

if(g==4) {

PA=(1-(e[n]/3))^A

PB=(e[n]/3)^B

}

probs[g+1]<-prod(PA,PB)

}

sp<-order(probs,decreasing=T)

rat<-probs[sp[1]]/probs[sp[2]]

locs[k,leels[i]]<-sp[1]-1

if(sum(locs[k,majcs[i]],locs[k,mincs[i]])<4) {

locs[k,leels[i]]<-5

}

}

}

write.table(locs,paste("ArenosaGTdata/",prefix[l],locus,".gts.out",sep=""),quote=F,row.names=F,col.names=F)

}

}

}

**##CLR test**

wsize=100

for(ch in 1:8) {

tr=subset(cr,cr[,1]==ch)

win=matrix(data=0,nrow=5,ncol=floor(length(tr[,1])/wsize))

s=1

e=wsize

for(i in 1:length(win[1,])-1) {

win[2,i]=mean(tr$V2[s:e])

win[3,i]=tr$V2[s]

win[4,i]=tr$V2[e]

win[5,i]=tr$V1[e]

tsfs=numeric(ng)

for(f in 1:ng) {

tsfs[f]=length(subset(tr[s:e,1],tr[s:e,39]==fq[f]))

}

p1=tsfs/sum(tsfs)

h0=p0^tsfs

h1=p1^tsfs

win[1,i]=2*(log(prod(h1))-log(prod(h0)))

#print(win[1,i])

s=e+1

e=e+wsize

}

if(ch==1) {

gwrc=win[5,]

gwr=win[1,]

gwsr=win[3,]

gwer=win[4,]

}

if(ch>1) {

gwrc=append(gwrc,win[5,])

gwr=append(gwr,win[1,])

gwsr=append(gwsr,win[3,])

gwer=append(gwer,win[4,])

}

#print(win)

}

dev.off()
